# Hair Growth-Supporting and Follicle-Protective Potential of a Botanical-Based Supplement Ingredient: In Vitro, Ex Vivo, and Molecular Docking Studies

**DOI:** 10.3390/biom16081207

**Published:** 2026-08-18

**Authors:** Adrián García, Andrea Cavagnino, Pau Navarro, Olivier Gouin, Cristina Guillem, Anaïs Bobier, Cristina Calabuig, Nuria Caturla

**Affiliations:** 1 Research and Development Department, Monteloeder SL, Miguel Servet 16, 03203 Elche, Alicante, Spain; adriangarcia@monteloeder.com (A.G.); paunavarro@monteloeder.com (P.N.); 2 OxiProteomics SAS, 2 Rue Antoine Etex, 94000 Créteil, Franceolivier.gouin@oxiproteomics.fr (O.G.); anais.bobier@oxiproteomics.fr (A.B.); 3 Textile and Cosmetic Industry Research Association (AITEX), Carretera Banyeres, 10, 03802 Alcoy, Alicante, Spain; cguillem@aitex.es (C.G.); cristina.calabuig@aitex.es (C.C.); 4 Department of Biotechnology, Universitat Politècnica de València (UPV), Camino de Vera s/n, 46022 Valencia, Valencia, Spain

**Keywords:** hair follicle, polyphenols, nutricosmetic, SRD5A2, β-catenin, IGF-1, Bcl-2, Ki67, environmental stress, protein carbonylation, human scalp explants, botanicals

## Abstract

Hair follicle homeostasis is influenced by hormonal pathways, the scalp microenvironment, and environmental stressors such as pollution, UV radiation, and oxidative stress. Elissara^®^, a polyphenol-enriched botanical ingredient, has shown benefits for scalp moisturization, barrier function, sebum regulation, and redness. Building on these scalp-level benefits, we investigated Elissara’s effects on follicular signaling, survival-associated biomarkers, oxidative damage, and androgen-related pathways as potential contributors to follicular health, using in silico, in vitro, and ex vivo models. Molecular docking (AutoDock Vina) of the main Elissara bioactives (oleuropein, hydroxytyrosol, verbascoside, carnosic acid, carnosol, and quercetin) identified SRD5A2 as a favorable predicted target, with individual binding energies ranging from −8.70 to −9.73 kcal/mol, approaching finasteride/dutasteride reference values. As an exploratory approach, simultaneous multi-ligand docking showed favorable global docking outputs for several targets, indicating that multiple bioactives could be structurally accommodated within complementary regions of the binding site. In human follicle dermal papilla cells, Elissara significantly increased BrdU incorporation to 245.70% of control at 0.002% and reduced SRD5A2 protein levels by 18.48% at 0.006%. In human scalp explants, Elissara at 200 µg/mL increased β-catenin, Bcl-2, and collagen IV under basal conditions and counteracted acute PM_2.5_/UVA-induced alterations in β-catenin, Ki67-positive cells, Bcl-2, IGF-1, collagen IV, and protein carbonylation. Together, these findings support the potential of Elissara as a promising nutricosmetic ingredient for supporting follicular resilience through multiple follicle-relevant pathways. Clinical studies assessing hair growth outcomes are needed to determine whether these preclinical findings translate into measurable benefits.

## 1. Introduction

Hair quality, and particularly hair loss, represents a major concern affecting millions of individuals worldwide and has a substantial psychosocial impact in both genders. Hair-related disorders are highly prevalent globally [1]. While androgenetic alopecia (AGA) accounts for the majority of chronic pattern hair loss, affecting approximately 50% of men and 25% of women by the age of 50 [2], acute conditions such as telogen effluvium, autoimmune disorders such as alopecia areata, and environmental or chemical damage also contribute to the growing burden of hair-related disorders [1].

Hair growth occurs in a continuous process characterized by four phases: anagen (growth), catagen (transition), telogen (resting), and exogen (shedding). In the human scalp, approximately 85% to 90% of hairs are in the anagen phase, while the rest are in catagen, telogen, or exogen [3]. Premature hair loss occurs when the normal hair growth cycle is disrupted, leading to excessive shedding and/or progressive follicular miniaturization [4]. Hair loss is a multifactorial condition driven by a complex interplay of genetic, hormonal, and inflammatory processes, with environmental and lifestyle factors further modulating its onset and progression [1]. Genetic predisposition is a major determinant in conditions such as AGA, the most common form of progressive hair thinning [2]. Androgens, particularly dihydrotestosterone (DHT), contribute to shortening of the anagen phase and progressive hair follicle miniaturization, while declining estrogen levels in women, particularly during peri- and postmenopause, may further contribute to hair thinning [2,5]. Two isoforms of 5α-reductase, type 1 and type 2, catalyze the conversion of testosterone to DHT, with type 2 being highly relevant in androgen-sensitive hair follicles and considered a key mediator of AGA [6]. DHT binding to androgen receptors in genetically susceptible follicles triggers a cascade that shortens the anagen phase, increases apoptosis, and progressively miniaturizes hair follicles [7].

Beyond genetic predisposition and hormonal drivers, current research highlights the critical role of external factors, collectively referred to as the exposome, in modulating hair health. These factors include environmental stressors such as airborne particulate matter, such as PM_2.5_, ultraviolet radiation (UVR), nutritional deficiencies, and chronic stress [8,9]. The scalp provides the biological microenvironment in which pre-emergent hair fibers develop, and its physiological integrity directly influences hair growth and hair quality [10].

Airborne pollutants and UVR induce excessive reactive oxygen species (ROS) production and activate the aryl hydrocarbon receptor (AhR) pathway, thereby triggering inflammatory and oxidative responses associated with follicular dysfunction [9,11,12]. Oxidative stress is currently recognized as a key contributor to both environmentally induced and age-related hair loss [13,14,15]. Individuals with active hair loss exhibit increased oxidative damage and reduced antioxidant defenses in scalp tissues compared with healthy subjects [16,17]. Hair follicles are particularly sensitive to this oxidative burden, which disrupts the balance of the hair growth cycle, prematurely shifting follicles from the anagen phase to catagen and telogen phases [14]. This disruption can lead to conditions such as telogen effluvium, where hair prematurely enters the shedding phase, ultimately resulting in increased hair shedding, hair thinning, and reduced hair density [14,15]. In addition, AhR overexpression has been detected in miniaturized follicles from women with female pattern hair loss, further supporting a mechanistic link between environmental exposure and follicular dysfunction [18].

Due to the limitations and side effects of pharmacological conventional hair loss treatments, natural and botanical-based approaches have gained interest as multi-target strategies with generally favorable tolerability. Extracts such as green tea, saw palmetto, ginseng, and pumpkin seed oil have been investigated for hair growth-supporting effects through mechanisms including 5α-reductase modulation, dermal papilla cell proliferation, antioxidant and anti-inflammatory activity, and anagen phase support [19,20,21,22].

In this respect, Elissara^®^ (formerly Zeropollution^®^) is a patented, polyphenol-enriched botanical combination composed of standardized extracts of *Lippia citriodora*, *Olea europaea*, *Sophora japonica*, and *Rosmarinus officinalis*. This proprietary formulation delivers a specific fingerprint of bioactive compounds, including verbascoside, oleuropein, hydroxytyrosol, quercetin, and rosemary-derived diterpenes (carnosol and carnosic acid) [23]. These phenolic constituents are widely recognized for their antioxidant, anti-inflammatory, photoprotective, and anti-pollution properties [24,25,26,27,28,29,30].

Previous studies demonstrated that this botanical combination can counteract UV- and pollution-induced oxidative stress, inflammation, and AhR-related responses [31]. Initially developed for protection against pollution-induced skin damage [31,32], Elissara subsequently showed clinically relevant scalp health benefits in a randomized, double-blind, placebo-controlled study involving 66 women. Oral supplementation with 250 mg/day for 12 weeks significantly improved scalp moisturization and reduced transepidermal water loss, sebum levels, and scalp redness [33]. Self-reported assessments revealed that 78.8% of participants reported improved hair quality, while 75.8% noted increased brightness and damage resistance [33].

Although these preliminary preclinical and clinical outcomes suggested a protective role of Elissara on scalp and hair condition, its direct effects on follicular biological pathways had not yet been elucidated. Interestingly, several individual bioactives present in this botanical blend have been associated with mechanisms relevant to hair follicle biology, including Wnt/β-catenin and growth factor-related signaling, dermal papilla cell protection, anti-inflammatory and antioxidant activity, reduced apoptosis, and follicular activation [34,35,36,37,38]. Therefore, the present study aimed to investigate whether Elissara could influence molecular and cellular pathways associated with hair follicle homeostasis and follicular resilience using an integrated in silico, in vitro, and ex vivo approach. First, molecular docking and multi-ligand simultaneous docking were performed with the main polyphenolic compounds present in the botanical blend to explore their potential interaction with protein targets involved in key mechanisms of hair growth regulation. Steroid 5α-reductase type 1 (SRD5A1) and steroid 5α-reductase type 2 (SRD5A2) were selected as androgen-related targets because they catalyze the conversion of testosterone into DHT, a central mediator of follicular miniaturization in AGA [6]. β-catenin was included as a core effector of Wnt signaling, which is essential for anagen initiation and follicular regeneration [39,40]. B-cell lymphoma 2 protein (BCL-2) was selected as a marker of anti-apoptotic signaling and follicular cell survival [41,42], whereas insulin-like growth factor 1 receptor (IGF-1R) was included because its signaling supports dermal papilla activity, matrix keratinocyte proliferation, and hair shaft elongation [43,44].

These computational analyses were complemented by in vitro experiments in human follicle dermal papilla cells (HFDPC), a key cellular population involved in hair cycle regulation and androgen-dependent follicular miniaturization [45]. In this model, Elissara was evaluated for its capacity to promote cell proliferation and modulate SRD5A2 levels. Finally, ex vivo human scalp explants were used to assess whether the ingredient could influence follicular biomarkers in a more physiologically relevant context. The selected biomarkers included β-catenin, Bcl-2, and IGF-1, reflecting growth-related signaling and follicular survival; Ki67, as a marker of proliferative activity [46]; collagen IV, as an indicator of basement membrane and extracellular matrix integrity [47,48]; and protein carbonylation, as a marker of oxidative damage [49,50]. These endpoints were evaluated under basal conditions and after PM_2.5_/UVA-induced environmental stress. This multi-level strategy aimed to determine whether the previously observed scalp-protective effects of Elissara could be mechanistically linked to direct support of follicular resilience and hair growth-related pathways.

## 2. Materials and Methods

### 2.1. Experimental Product

The test item was a patented botanical ingredient protected by the ES2689105B8 patent and related international patent family WO2019211501A1 [23], commercially available food supplement ingredient under the registered name Elissara^®^ (trademark family Zeropollution^®^) supplied by Monteloeder SL. (Suannutra group, Elche, Spain). The ingredient is a blend of four standardized botanical extracts: *Rosmarinus officinalis* leaf extract standardized in diterpenes, *Olea europaea* leaf extract standardized in oleuropein and hydroxytyrosol, *Lippia citriodora* leaf extract standardized in verbascoside and *Sophora japonica* extract standardized in quercetin. In total, *w*/*w* this blend comprises a minimum content of the following phenols: diterpenes (sum of carnosic acid and carnosol) 4.5%; oleuropein 4.5%; hydroxytyrosol 1.5%; verbascoside 6.8%; and flavonoids as quercetin minimum 3.7%. These main compounds were identified and quantified by HPLC-DAD analysis, comparing retention time and UV spectra of the peaks in samples with those of authentic standards as previously described [32]. As Elissara is a pre-established, patented, and commercially available standardized botanical blend, the present study was designed to evaluate the biological activity of the complete ingredient rather than to optimize the formulation ratio or compare isolated constituents and pairwise combinations.

### 2.2. In Silico Molecular Docking Analysis

#### 2.2.1. Active Compounds and Ligand Preparation

The active compounds evaluated in this in silico study were the main bioactive constituents associated with the Elissara botanical blend. The selected compounds were verbascoside, quercetin, hydroxytyrosol, oleuropein, carnosic acid, and carnosol.

Canonical Simplified Molecular Input Line Entry System (SMILES) strings, International Chemical Identifier Keys (InChIKeys), and three-dimensional structure-data file (SDF) structures were retrieved from PubChem for each compound: verbascoside, compound identifier (CID) 5281800; quercetin, CID 5280343; hydroxytyrosol, CID 82755; oleuropein, CID 5281544; carnosic acid, CID 65126; and carnosol, CID 442009.

In addition to the botanical test compounds, target-specific reference control molecules were included to support methodological comparison. Venetoclax, also known as ABT-199, PubChem CID 49846579, was selected as the reference compound for B-cell lymphoma 2 protein (BCL-2), based on its established binding mode as a BH3-mimetic at the hydrophobic BH3-binding groove [51]. For β-catenin, iCRT14, PubChem CID 5288209, was used as the primary reference because it has been described as a small-molecule inhibitor of the β-catenin/TCF transcriptional interaction [52]. The β-catenin/TCF4 interaction interface was further supported by structural information from Graham et al. [53].

For insulin-like growth factor 1 receptor (IGF-1R), an ATP-competitive benzimidazole-class inhibitor of the IGF-1R kinase domain was used as the reference compound, based on its compatibility with the ATP-binding cleft of the receptor structure used for docking [54]. For steroid 5α-reductase type 1 (SRD5A1) and steroid 5α-reductase type 2 (SRD5A2), finasteride, PubChem CID 57363, and dutasteride, PubChem CID 6918296, were included as pharmacological reference inhibitors of the dihydrotestosterone (DHT) biosynthesis pathway [55]. β-Sitosterol, PubChem CID 222284, was also included as a natural reference for both 5α-reductase isoforms [56].

All botanical compounds and reference control molecules were processed using the same ligand preparation workflow. Ligand structures were prepared using AutoDock Tools 1.5.7. Non-polar hydrogens were merged, Gasteiger–Marsili partial charges were assigned, and all rotatable bonds were defined as active. The prepared ligand structures were subsequently exported in Protein Data Bank, Partial Charge and Atom Type (PDBQT) format and used for molecular docking without additional geometry optimization. Ligand protonation and tautomeric states were not independently optimized for a defined pH; the molecular states present in the structures retrieved from PubChem were retained during preparation. This represents a limitation of the docking workflow because alternative ionization or tautomeric states may affect predicted poses and scores.

#### 2.2.2. Molecular Target Selection, Receptor Preparation, and Docking-Site Definition

Molecular targets were selected based on published evidence linking the selected proteins to hair follicle biology, androgen metabolism, apoptosis regulation, Wnt/β-catenin signaling, and growth factor-mediated follicular activity. Complementary bioinformatic resources, including GeneCards [57], the Human Protein Atlas [58], STRING [59], STITCH [60], and the Reactome Pathway Browser [61], were consulted to corroborate gene function, tissue and cell-type expression, pathway context, and known protein- or compound-association networks.

Five molecular targets were selected to represent distinct regulatory nodes relevant to hair follicle biology and androgen-related follicular regulation: steroid 5α-reductase type 2 (SRD5A2), steroid 5α-reductase type 1 (SRD5A1), B-cell lymphoma 2 protein (BCL-2), β-catenin, and insulin-like growth factor 1 receptor (IGF-1R).

Experimental structures were obtained from the Protein Data Bank when available. For SRD5A1, an AlphaFold-predicted structural model was used due to the lack of a suitable experimentally resolved human structure. Receptor structures were prepared using AutoDock Tools 1.5.7. Water molecules, non-relevant heteroatoms, and co-crystallized ligands were removed, except when used as structural references for binding-site definition. Polar hydrogens were added, Kollman charges were assigned, and the prepared receptor structures were exported in Protein Data Bank, Partial Charge and Atom Type (PDBQT) format.

Binding-site definition was based on crystallographic ligand positions, the literature-reported active-site annotations, and complementary structure-based pocket analysis using DoGSiteScorer [62] through the ProteinsPlus web server (https://proteins.plus/, accessed on 5 March 2026). DoGSiteScorer was used to evaluate whether the selected docking regions corresponded to structurally defined and chemically plausible binding pockets. This analysis was used as methodological support for binding-site selection and grid-box placement, but it was not treated as an independent quantitative endpoint.

Docking grid boxes were centered on the known or predicted binding site of each receptor. For receptors with co-crystallized ligands, the grid center was defined according to the ligand-binding region observed in the crystallographic structure. For SRD5A1, the docking region was defined using structural alignment and literature-supported residues associated with the steroid-binding cavity. A grid spacing of 0.375 Å was used throughout the study.

For multi-ligand simultaneous docking (MLSD), the same grid centers used in the individual docking protocol were retained, and the search box was expanded by 10 Å in each direction to allow accommodation of multiple ligands within the binding region. This expansion was intended to cover the original pocket and adjacent subcavities while avoiding a shift in the search towards unrelated surface regions.

The receptor structures and grid box parameters used for each molecular target are summarized in Table 1.

#### 2.2.3. Molecular Docking and Interaction Analysis

Molecular docking simulations were performed using AutoDock Vina 1.1.2 implemented in PyRx 1.2 with a rigid-receptor approach and ligand flexibility allowed through rotation around non-terminal, non-ring bonds. For each ligand–target pair, five independent docking runs were performed using standardized settings (exhaustiveness 8, nine output poses, energy range 4 kcal/mol). The three lowest-scoring poses across the complete output set were retained for descriptive analysis. AutoDock Vina docking scores expressed in kcal/mol provide a computational estimate of relative binding favorability for pose ranking and comparison within the same docking protocol, but they should not be interpreted as experimentally determined binding free energies or direct quantitative measures of affinity. The same protocol was applied to all botanical compounds and reference control molecules.

The use of a fixed receptor structure was particularly appropriate for the initial assessment of poses within binding regions previously defined using co-crystallized ligands, functional annotations, and cavity analysis. Consequently, the results were interpreted as estimates of geometric compatibility with the structural conformation used and not as a complete representation of protein dynamics or the induced fit associated with binding.

MLSD was used to evaluate the collective binding behavior of the bioactive compounds present in Elissara within each selected binding site. In this approach, the full set of Elissara-representative compounds was docked simultaneously to assess whether the ligands could coexist within the same binding pocket, occupy complementary subpockets, preserve relevant protein–ligand interactions, or instead compete for overlapping regions and induce mutual displacement. These simulations were interpreted qualitatively as exploratory structural assessments rather than as quantitative affinity measurements or evidence of biological activity, additive affinity, or synergistic interactions.

Post-docking interaction analysis was also performed. Residues located within 4.5 Å of each ligand were identified, and interactions were classified as conventional hydrogen bonds, hydrophobic contacts, π-alkyl interactions, alkyl interactions, or van der Waals interactions. Conventional hydrogen bonds were defined by a donor-acceptor distance of ≤3.5 Å and a donor–hydrogen–acceptor angle of ≥120°.

Two-dimensional interaction diagrams of representative complexes were generated using PoseView through the ProteinsPlus web server. These diagrams supported residue-level interpretation and were complemented by three-dimensional visual inspection. Residues recurring in at least two of the three retained poses were considered consistently involved in ligand recognition and were prioritized in the results. When available, interaction profiles were compared with previously described pharmacophoric residues for each target. For MLSD, poses were visually inspected to determine whether ligands could coexist within the same binding site in a chemically plausible pose. Actives were considered structurally compatible when they occupied complementary subpockets, preserved favorable interactions, and showed no relevant steric overlap. Combinations were considered competitive or inconclusive when ligands overlapped within the same binding region, displaced one another, or adopted unrealistic orientations. Multi-ligand docking scores were not interpreted as individual affinities or as the sum of single-ligand docking energies; instead, results were evaluated by integrating docking score, spatial coherence, absence of steric clash, conservation of relevant contacts, and occupation of compatible subpockets.

### 2.3. In Vitro Efficacy Study in HFDPC

#### 2.3.1. Cell Culture Conditions

Human Follicle Dermal Papilla Cells (HFDPC) (C-12071, manufactured by PromoCell GmbH, Heidelberg, Germany) were cultured according to the supplier’s instructions under standard conditions at 37 °C in a humidified atmosphere with 5% CO_2_ until reaching 80–90% confluence. Cells were seeded in 96-well plates (VWR International, Radnor, PA, USA) and allowed to adhere overnight prior to treatment.

#### 2.3.2. Reagents and Test Samples

PBS, PrestoBlue reagent, DMSO, ethanol, penicillin-streptomycin, and Dermal Cell Basal Medium and trypsin were obtained from VWR Chemicals (Leuven, Belgium). The Cell Proliferation ELISA BrdU (colorimetric) kit and Human SRD5A2 ELISA kit were obtained from Antibodies (Cambridge, UK). Epidermal growth factor (EGF) was obtained from Sigma-Aldrich, Merck (St. Louis, MO, USA), finasteride was purchased from Thermo Fisher Scientific (Waltham, MA, USA), and testosterone was purchased from IBL International GmbH (Hamburg, Germany).

#### 2.3.3. Cytotoxicity Assay

Cytotoxicity was assessed according to ISO 10993-5:2009 [63]. HFDPC were treated with eight concentrations (0.0004%, 0.0008%, 0.0015%, 0.003%, 0.00625%, 0.0125%, 0.025%, and 0.05%) of Elissara for 24 h, with five technical replicates per concentration. After treatment, cells were washed with PBS and incubated with PrestoBlue reagent for 3 h at 37 °C. Absorbance was measured at 570 and 600 nm using a microplate reader (BMG LABTECH GmbH, FLUOstar Omega, Ortenberg, Germany). Raw absorbance values were exported from the instrument software, and cell viability was calculated relative to untreated control cells. Concentrations maintaining viability above 70% were considered non-cytotoxic and were selected for subsequent studies.

#### 2.3.4. Proliferation Assay

Cellular proliferative potential was evaluated through the incorporation of 5-bromo-2′-deoxyuridine (BrdU) during DNA synthesis, using the Cell Proliferation ELISA, BrdU (colorimetric) kit (Antibodies, Cambridge, UK). This method quantifies cell proliferation based on DNA replication activity [64]. HFDPC were treated for 24 h with two non-cytotoxic concentrations of Elissara, with the aim of evaluating biological activity rather than establishing a complete dose–response curve. After treatment, BrdU was added and incubated according to the manufacturer’s instructions. DNA synthesis was quantified by optical density measurement. Untreated cells were used as a negative control, and treated cells with Epidermal Growth factor (EGF) at 10 nM were used as a positive control. Two independent experiments were performed, each including four technical replicates per condition. Cell proliferation was expressed as a percentage relative to the negative control using the following equation: % proliferation = (sample/C−) × 100, where “C−” corresponds to the mean absorbance of untreated cells.

#### 2.3.5. Effect on 5α-Reductase Type II (SRD5A2) Levels

Modulation of Elissara on 5α-reductase type II protein levels was evaluated using a Human SRD5A2 ELISA kit (Antibodies, Cambridge, UK). HFDPC were treated for 24 h with two non-cytotoxic concentrations of Elissara. HFDPC treated with testosterone (120 µM) were used as the androgen-stimulated control, and finasteride (50 µM) was included as a reference control. After incubation, cells were collected, lysed, and SRD5A2 levels were quantified according to the manufacturer’s instructions. Results were normalized to cell viability. Two independent experiments were performed, each including four technical replicates per condition. The percentage of reduction was calculated relative to the testosterone-treated control using the following equation: % reduction = [(T − sample)/T] × 100, where T corresponds to the mean signal of the testosterone control.

#### 2.3.6. Statistical Analysis

Data were analyzed using JAMOVI (Version 2.5, Sydney, Australia). Outliers were identified using the interquartile range (IQR) method and excluded prior to statistical analysis when considered technical or instrumental deviations. For each assay, the final number of analyzed replicates is indicated in the corresponding Section 3 or figure legend. Data normality was assessed using the Shapiro–Wilk test, and homogeneity of variances was evaluated using Levene’s test. For both proliferation and SRD5A2 assays, differences between groups were analyzed using one-way ANOVA, followed by Tukey’s post hoc test for multiple comparisons. Statistical significance was set at *p* < 0.05.

### 2.4. Protective and Regenerative Effects of Elissara in Human Living Scalp Explants

The ex vivo study evaluated biomarkers associated with hair follicle growth, cycling, extracellular matrix integrity, survival, and oxidative damage, namely β-catenin, Ki67, Collagen IV, Bcl-2, IGF-1, and protein carbonylation.

#### 2.4.1. Ex Vivo Human Scalp Explant Culture and Experimental Design

Human scalp explants were obtained, with informed consent, from lifting surgery of a 53-year-old Caucasian female donor without a clinical hair-loss diagnosis (Ref. BIOP-1-2025-12-16), and kept alive on inert grids in standard 12-well plates supplied by Corning SAS (Boulogne-Billancourt, France), containing OxiProteomics^®^ culture medium in a humidified atmosphere with CO_2_ (5% *v*/*v*) at 37 °C. The study was conducted by OxiProteomics SAS (Créteil, France) in accordance with the Declaration of Helsinki and involved the use of biological samples derived from human subjects, in accordance with regulations and ethical standards, approved by the French Ministry of Higher Education and Research (AC-2022-5147, last approval date 3 January 2023). Upon receipt, the test material, Elissara (Monteloeder S.L., Elche, Spain), was stored according to the sponsor’s instructions until use.

Scalp explants were distributed into 4 experimental groups (*n* = 3 explants per group) and grouped according to the treatment plan (Table 2).

The culture medium was renewed every 24 h. The active ingredient (Elissara) was diluted in culture medium to a final concentration of 200 µg/mL and maintained in contact with the explants for 24 h; this treatment was repeated for three consecutive days.

The 200 µg/mL concentration was selected based on the dose previously shown to be non-cytotoxic and well tolerated in skin explant studies with the same botanical formulation, allowing direct comparability with earlier preclinical findings [31], rather than to define the optimal effective concentration in scalp explants.

For stress conditions, explants were topically treated on Day 4 with a solution (0.375 μg/cm^2^) containing urban ultra-fine dust (PM_2.5_-like; ERM-CZ110, certified European Reference Material) for 30 min and then irradiated with UV-A light (λ = 365 nm; 6 J/cm^2^) using the OxiProteomics^®^ irradiation system. Two hours after stress exposure, the medium was replaced with fresh medium without the active ingredient. Twenty-four hours after the last treatment under baseline conditions, or 24 h after stress exposure under stressed conditions, explants were collected, embedded in OCT (Cryomatrix™, Thermo Fisher Scientific, Asnières-sur-Seine, France), and cryopreserved at −80 °C until analysis. To note that this PM_2.5_/UVA challenge was designed as an acute environmental stress model to induce measurable damage in scalp explants, rather than to fully reproduce chronic real-life scalp exposure, which involves repeated low-dose exposure to complex pollutant mixtures and solar radiation over time.

#### 2.4.2. In Situ Visualization

Scalp sections (5 μm thickness) were obtained using a Leica CM1950 cryostat (Leica Biosystems, Wetzlar, Germany) and subsequently fixed and permeabilized with Oxiproteomics^®^ solution (OxiProteomics SAS, Créteil, France). In situ protein carbonylation was detected using a fluorescent probe (Ex = 647 nm/Em = 650 nm) which specifically binds to carbonyl moieties, as previously described [65]. For immunodetection, non-specific binding sites were blocked with 3% bovine serum albumin (BSA) in phosphate-buffered saline (PBS, pH 7.4). Tissue sections were then incubated with the corresponding primary antibodies diluted in 3% BSA/PBS. After washing with PBS to remove excess primary antibody, sections were incubated for 1 h with fluorophore-conjugated secondary antibody. The cellular nuclei were labelled with DAPI (4′,6-diamidino-2-phenylindole). Finally, the antibody and DAPI excess were removed with a sequence of washing steps with PBS.

The primary antibodies used were rabbit anti-β-catenin (Abcam, ab305261, Cambridge, UK), rabbit anti-Ki67 (Abcam, ab92742, Cambridge, UK), rabbit anti-collagen IV (Abcam, ab6586, Cambridge, UK), rabbit anti-IGF-1 (Abcam, ab9572, Cambridge, UK), and rabbit anti-Bcl-2 (Abcam, ab59348, Cambridge, UK). A goat anti-rabbit Alexa Fluor 647 secondary antibody was used for fluorescent detection (Invitrogen, A21244, Carlsbad, CA, USA).

#### 2.4.3. Image Analysis and Statistics

Fluorescent images (16-bit, TIFF format) were acquired across the full dynamic range of the specific signal using an epifluorescence microscope (EVOS M5000 or M7000 Imaging System, Thermo Fisher Scientific, USA) and analyzed with ImageJ (software version 1.53) [66]. For β-catenin, collagen IV, Bcl-2, IGF-1, and carbonylation, signal intensity was quantified by integrating fluorescence signal over threshold and normalizing it to the evaluated hair follicle surface area. For Ki67, proliferative activity was expressed as the percentage of Ki67-positive cells relative to the total number of nuclei within the analyzed follicular region.

For each biomarker, values were normalized to the untreated control group, which was set at 100%, and data were expressed as mean ± standard deviation (SD) for *n* = 3 explants per group. Under stress conditions, the relative effect of Elissara was expressed both as percentage change compared with the stress group and, when applicable, as recovery of the stress-induced alteration. Protective efficacy or recovery of the stress-induced loss was calculated as: Protective efficacy (%) = [([ELS + STRESS] − STRESS)/(CTRL − STRESS)] × 100. For biomarkers increased by stress, such as protein carbonylation, protective efficacy was calculated as the percentage reduction in the stress-induced increase using the formula: Protective efficacy (%) = [(STRESS − [ELS + STRESS])/(STRESS − CTRL)] × 100.

Statistical analyses were carried out using GraphPad software (version 10.0.3, GraphPad Software, La Jolla, CA, USA) with an unpaired *t*-test using Welch’s correction. Baseline comparisons were performed versus the control group, whereas protective-effect comparisons were performed versus the stress group, using a 95% confidence interval. The explant was considered the experimental unit. When multiple images or follicular regions were analyzed from the same explant, values were averaged to obtain one value per explant before statistical comparison, thereby avoiding pseudo-replication. No outliers were identified or excluded from the ex vivo datasets based on the IQR method. Values of *p* < 0.05 were considered statistically significant, whereas 0.05 ≤ *p* < 0.10 was reported as a statistical trend when indicated.

To address the risk of multiple comparisons across the ex vivo biomarker panel, a post-hoc Benjamini–Hochberg false discovery rate (BH-FDR) correction was applied as a sensitivity analysis across the 17 pairwise comparisons performed for the six biomarkers evaluated.

Raw *p*-values are reported in Section 3 and figure legends for consistency with the predefined pairwise comparisons, whereas BH-adjusted *p*-values were used to assess the robustness of the findings.

## 3. Results

### 3.1. In Silico Molecular Docking Analysis Results

Individual botanical compounds showed moderate to favorable docking scores across multiple targets (Table 3).

**Figure 1:**
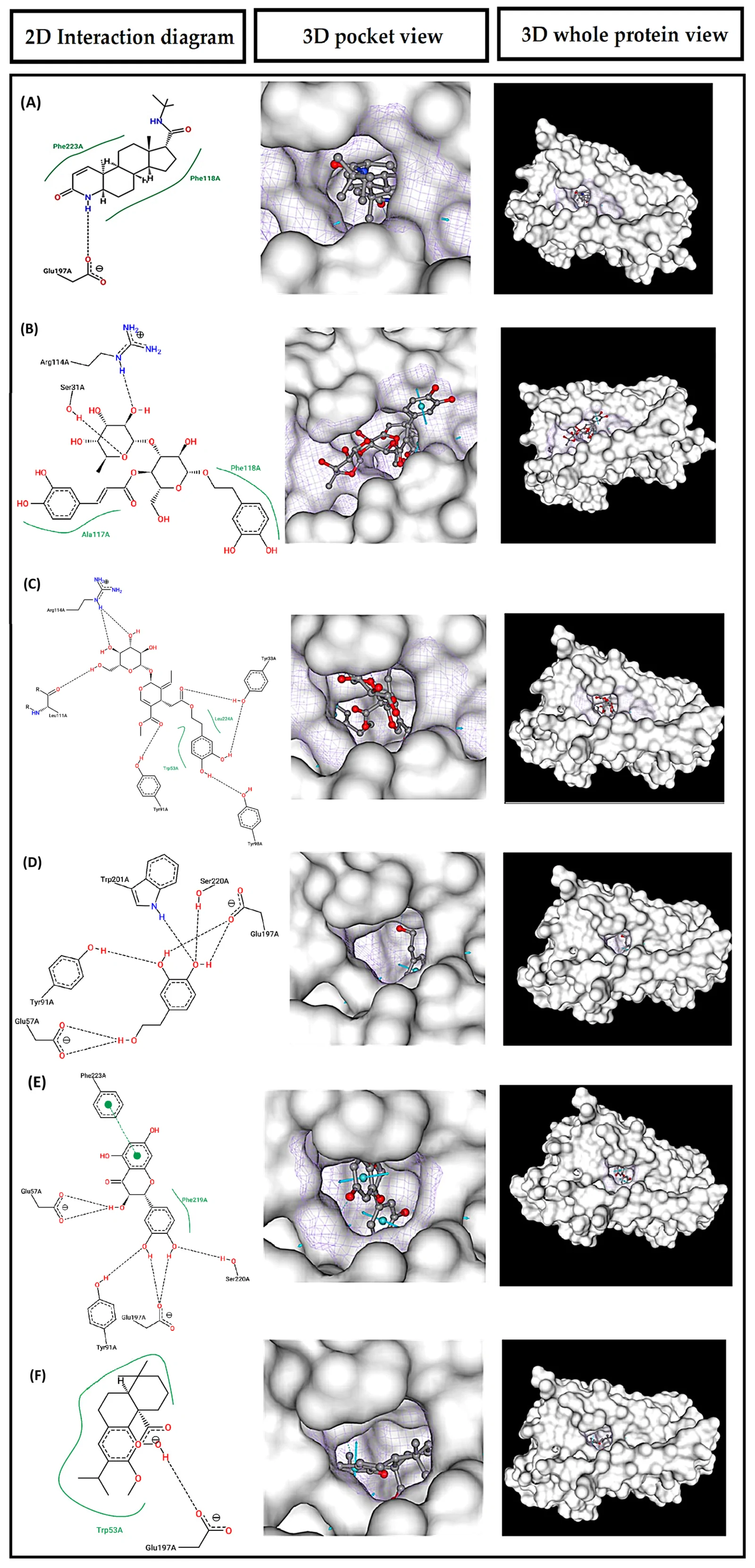
Single-ligand molecular docking results for finasteride and selected Elissara bioactive compounds against SRD5A2. Rows correspond to finasteride (**A**), verbascoside (**B**), oleuropein (**C**), hydroxytyrosol (**D**), quercetin (**E**), and carnosic acid (**F**). For each ligand, the left column shows the two-dimensional interaction diagram, the middle column shows the predicted docking pose within the SRD5A2 binding pocket, and the right column shows the ligand position in the whole-protein surface view. 2D interaction diagrams were generated using PoseView. In these diagrams, directed interactions between the ligand and protein residues are represented by dashed lines, whereas hydrophobic contacts are shown as green curved splines near the hydrophobic regions of the ligand and the interacting residues. Atoms are displayed according to the PoseView color convention, and residue labels indicate the receptor residues involved in the predicted interactions. The panels show representative predicted docking poses obtained from the molecular docking analysis.

The most favorable individual profiles were observed for the two 5α-reductase isoforms, which are directly involved in androgen metabolism and DHT biosynthesis. For steroid SRD5A2, all compounds, except hydroxytyrosol, exhibited binding energies between −8.70 and −9.73 kcal/mol, approaching but not surpassing the reference inhibitor dutasteride (−11.93 kcal/mol). Oleuropein showed the most favorable binding energy at this target (−9.73 kcal/mol), followed by verbascoside, carnosol, quercetin, and carnosic acid. Several of these compounds interacted with residues located within the SRD5A2 catalytic environment. Notably, verbascoside and carnosic acid contacted Glu197, while Glu57, Arg94, Trp53, Phe118, Phe223, and Leu224 were recurrently involved in the binding profiles of two or more compounds. The interaction patterns included hydrogen-bonding and hydrophobic contacts within the catalytic pocket, supporting the structural compatibility of the main Elissara bioactives with this target. As an example of the molecular docking analyses performed, Figure 1 shows the predicted interaction of finasteride and selected bioactive compounds present in Elissara with SRD5A2. The predicted docking poses show their accommodation within the SRD5A2 binding pocket, together with their corresponding 2D amino acid interaction profiles.

At SRD5A1, verbascoside reached −10.37 kcal/mol, a value close to dutasteride (−10.45 kcal/mol). Verbascoside showed an extensive predicted interaction network involving Arg232, Tyr102, Arg176, Lys110, Lys184, Asn198, Glu202, Glu60, Asp169, Tyr199, and Tyr240, while oleuropein engaged Trp56 and Tyr199, residues associated with the steroid-binding cavity. Due to the lack of an SRD5A1 crystal structure, docking was performed using the AlphaFold-predicted model. Therefore, SRD5A1 results should be interpreted with caution.

Regarding β-catenin, verbascoside, oleuropein, quercetin, carnosic acid, and carnosol showed binding energies ranging from −6.00 to −6.60 kcal/mol, slightly weaker than the iCRT14 control (−6.80 kcal/mol). The leading compounds interacted with residues located within or near the T-cell factor 4 (TCF4)-binding interface, particularly Arg386 and Asn387. Oleuropein showed the broadest interaction footprint, involving Arg386, Asn387, Asp390, Ser425, Gly422, Asn426, Lys354, Thr418, and Asp459, whereas quercetin showed a more compact interaction pattern involving Glu462, Asn387, Arg386, Asp390, and Ser389.

For BCL-2, verbascoside and oleuropein showed the most favorable scores, −8.20 and −7.13 kcal/mol, respectively, although both remained weaker than venetoclax (−10.60 kcal/mol). Verbascoside consistently interacted with residues within the BH3-binding groove, including Asp100, Phe101, Tyr105, Met112, Asn140, Gly142, and Ala146. These residues contributed to a mixed interaction profile involving polar contacts and hydrophobic/aromatic stabilization within the BH3 groove. Other compounds maintained contacts with residues distributed along the hydrophobic groove, including Tyr105, Phe101, Gly142, Tyr199, and Ala97.

At IGF-1R, quercetin exhibited the most favorable individual docking score (−8.00 kcal/mol) compared with the benzimidazole reference compound (−7.53 kcal/mol). Its predicted binding mode involved residues within the kinase-domain environment, including Met1052, Met1126, Met1112, Asp1056, and Val983.

Of all the Elissara actives, hydroxytyrosol showed the weakest binding energies across all targets, ranging from −4.57 to −5.97 kcal/mol. This result is consistent with its smaller molecular size, 154.16 Da, and reduced interaction surface area compared with the other botanical constituents.

When simultaneous multi-ligand docking was performed, global docking outputs of −15.65, −15.73, −16.44, and −16.43 kcal/mol were obtained for SRD5A2, β-catenin, BCL-2, and IGF-1R, respectively and were numerically more negative than those obtained for the corresponding single-reference ligands. However, these values should be interpreted as an exploratory indicator of structural compatibility rather than as a quantitative measure of binding affinity or biological synergy. Together, these findings suggest a structurally compatible multi-ligand arrangement among the Elissara bioactive compounds within selected binding sites, consistent with the occupation of compatible adjacent subpockets and preservation of relevant protein–ligand interactions.

For BCL-2, the simultaneous multi-ligand docking assembly broadly occupied the BH3-binding groove and involved residues such as Arg143, Asn140, Asp100, Met112, Ala146, Tyr199, Gly142, Tyr105, and Gln96. At SRD5A2, the simultaneous pose suggested compatible occupation of the predicted catalytic pocket region, with polar contacts involving Arg114, Gln56, Asn102, Arg103, and Arg227, together with additional interactions involving Ala62 and Thr126. Regarding the β-catenin/TCF4 interface, simultaneous multi-ligand docking showed coherent occupation of the interface region, with contacts involving Asn387, Glu462, and Arg469. For IGF-1R, the compound assembly contacted residues within the kinase pocket, including Asp1056, Met1112, Val1089, Glu1030, Glu1193, Lys1120, and Cys1029. The exception was SRD5A1, where the simultaneous presence of all compounds produced a less favorable global docking output of −7.05 kcal/mol. Although contacts with Arg176, Asp169, Asn198, Tyr199, Phe118, and Leu229 were observed, the complete ligand assembly did not appear to adopt the same favorable spatial organization observed for the other targets. This result suggests less favorable co-accommodation of the complete compound set within the predicted SRD5A1 pocket, possibly due to geometric constraints or overlapping ligand positions.

Overall, individual docking identified the 5α-reductase isoforms, particularly SRD5A2, as the most favorable targets for Elissara bioactives, whereas simultaneous multi-ligand docking highlighted BCL-2, SRD5A2, β-catenin, and IGF-1R as binding sites capable of accommodating the complete compound set in structurally coherent arrangements.

### 3.2. In Vitro Efficacy Study in Human Follicle Dermal Papilla Cells

Cytotoxicity evaluation showed that HFDPC viability remained above the predefined non-cytotoxicity threshold at all tested concentrations up to 0.0125% of Elissara^®^ (Supplementary Figure S1). In contrast, 0.025% and 0.05% reduced viability below 70% and were therefore considered cytotoxic according to ISO 10993-5:2009 [63]. Based on these results, 0.002% and 0.006% were selected for subsequent proliferation and SRD5A2 assays.

#### 3.2.1. Cell Proliferation Assay

**Figure 2:**
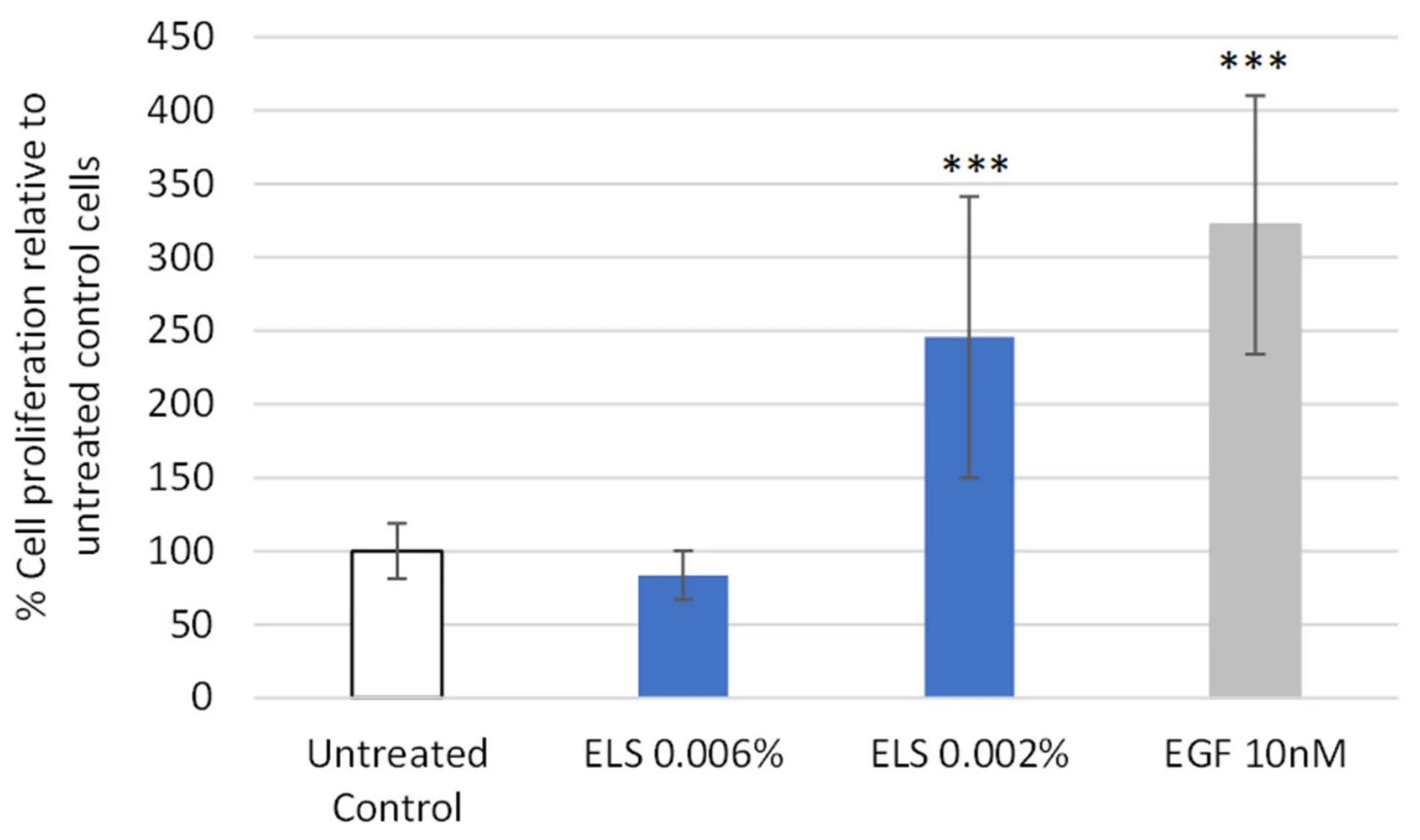
Effect of Elissara on cell proliferation in human dermal papilla cells (HFDPC). Bar graphs represent cell proliferation levels, normalized to untreated control, in HFDPC after treatment with Elissara (ELS) at 0.002% and 0.006% or epidermal growth factor (EGF) at 10 nM for 24 h. Data are presented as mean ± SD. A single outlier in the 0.002% group was identified and removed using the IQR method before statistical analysis. Asterisks indicate significant differences vs. untreated control: *** *p* < 0.001.

Dermal papilla cells play a central role in the regulation of hair follicle growth by controlling keratinocyte proliferation and hair cycle progression [45]. Therefore, stimulation of dermal papilla cell proliferation is considered a relevant in vitro indicator of hair growth-supporting potential. In this context, we studied whether Elissara could promote the proliferation of HFDPC. The results of this study are shown in Figure 2.

One extreme value in the 0.002% group was excluded according to the predefined IQR criteria before the statistical analysis. Treatment with Elissara^®^ at 0.002% resulted in a marked increase in cell proliferation, reaching 245.70 ± 95.73% (*n* = 7), which was significantly higher than the negative control (*p* < 0.001). Although no statistically significant difference was observed between the 0.002% Elissara group and the positive control (EGF; 322.17 ± 87.95%, *p* < 0.001 vs. negative control), this comparison should be interpreted with caution due to the relatively high variability within the treatment group. In contrast, treatment at 0.006% did not increase proliferation, showing values of 83.66 ± 16.66% (*n* = 8), with no significant differences compared to the negative control (*p* > 0.05).

#### 3.2.2. Effect of Elissara on 5α-Reductase Type II (SRD5A2)

Steroid 5α-reductase 2 (SRD5A2) plays a central role in the pathogenesis of androgenetic alopecia (AGA), or common hair loss, by converting testosterone into the more potent androgen dihydrotestosterone (DHT) within hair follicles. Excessive DHT leads to follicle miniaturization and, eventually, hair thinning [67].

**Figure 3:**
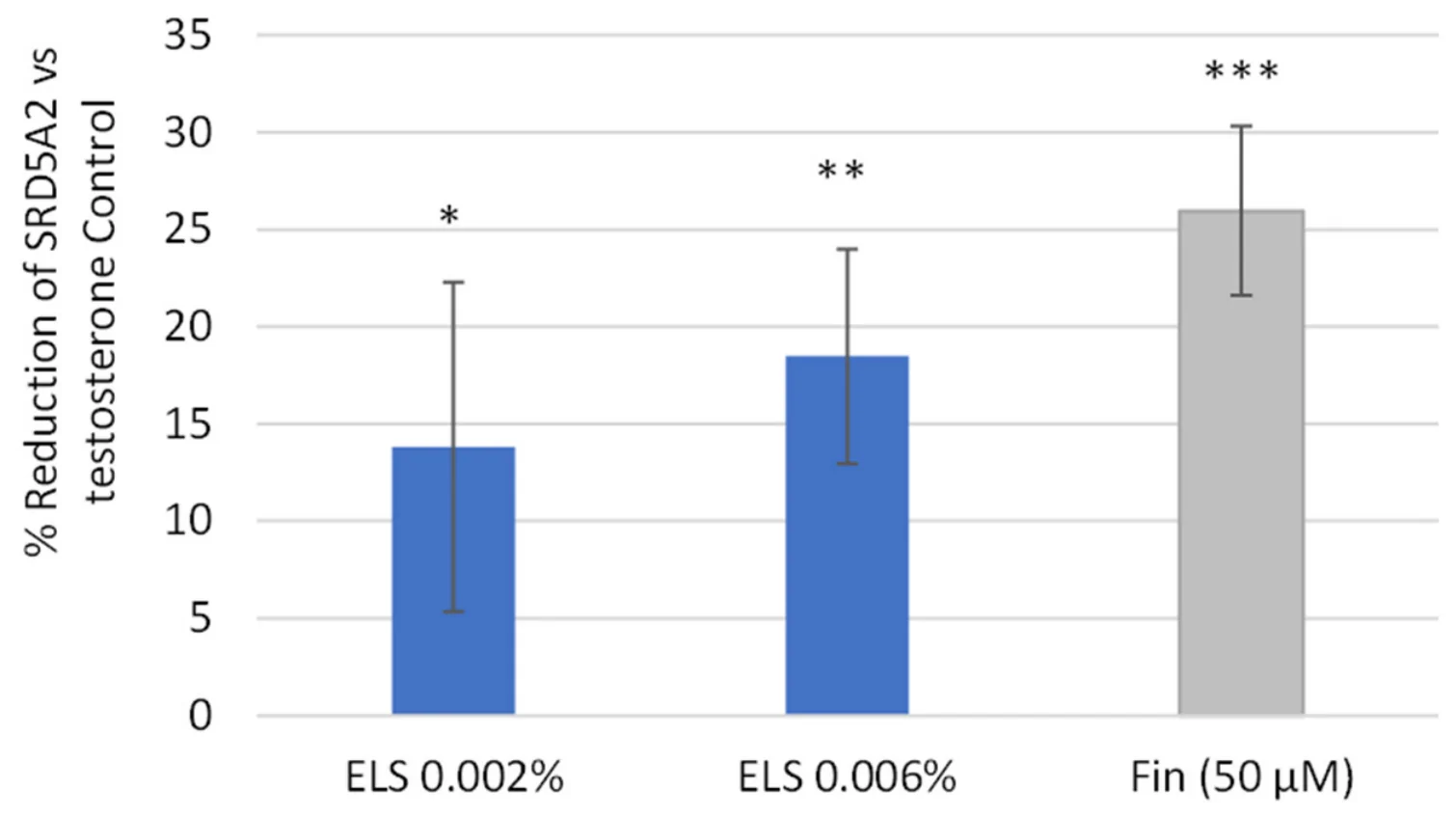
Effect of Elissara on 5α-reductase type II (SRD5A2) protein levels in human dermal papilla cells. Bar graphs represent SRD5A2 levels, normalized to testosterone-treated control, in human HFDPC after treatment with Elissara (ELS) at 0.002 and 0.006% and finasteride (Fin) at 50 µM for 24 h. Data are presented as mean ± SD. Asterisks indicate significant differences vs. testosterone control as follows: * *p* < 0.05, ** *p* < 0.01, *** *p* < 0.001.

As shown in Figure 3, the percentage reduction of SRD5A2 levels for each sample is expressed relative to the testosterone-treated control. HFDPC treated with Elissara showed a significant reduction of SRD5A2 levels at both tested concentrations. At 0.002%, Elissara reduced SRD5A2 by 13.82 ± 8.47% compared to testosterone-treated cells (*p* < 0.05). At 0.006%, this reduction increased to 18.48 ± 5.51% (*p* < 0.01), suggesting a concentration-dependent trend under the tested conditions.

Although finasteride showed the greatest reduction (−25.97 ± 4.37%), the difference between finasteride and Elissara at 0.006% did not reach statistical significance under the conditions tested (*p* > 0.05).

Overall, these results indicate that Elissara reduced SRD5A2 protein levels in HFDPC, suggesting a potential modulation of androgen-related pathways under the experimental conditions tested.

### 3.3. Ex Vivo Assessment of the Protective and Regenerative Effects of Elissara in Human Scalp Explants

#### 3.3.1. Effect of Elissara on β-Catenin Levels

**Figure 4:**
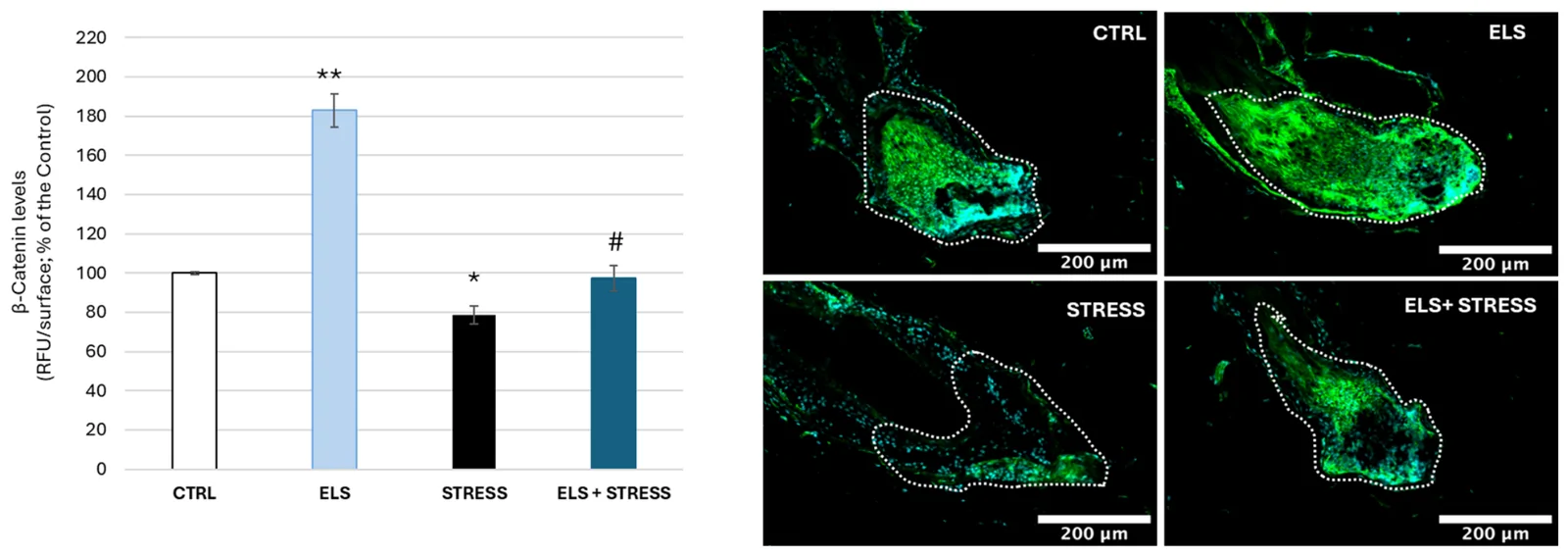
Effect of Elissara on β-catenin levels. Bar graphs represent β-catenin levels, normalized to untreated control (CTRL), in the hair bulb region of human scalp explants after treatment with Elissara (ELS) at 200 µg/mL for three consecutive days, in the absence or presence of environmental stress. Data are presented as mean ± SD of 3 explants per condition. Asterisks indicate significant differences vs. CTRL: * *p* < 0.05, ** *p* < 0.01. Hash symbols indicate significant differences vs. STRESS: # *p* < 0.05. Representative images show in situ visualization of β-catenin signal in hair follicles (green), superimposed on nuclear staining (DAPI, cyan). Images were acquired with a 10× objective. Regions of interest (ROI) are delineated with dotted lines. Scale bar = 200 µm.

β-catenin, a key effector of the Wnt signaling pathway, is essential for initiating and maintaining the anagen phase in hair follicles by activating stem cells and regulating follicular morphogenesis [39,40,68,69]. Environmental stressors, such as UVR and pollutants, can impair follicular homeostasis through oxidative stress and inflammatory responses, potentially affecting growth-associated signaling pathways such as Wnt/β-catenin [9]. This study evaluated both the ability of Elissara to increase β-catenin levels under basal conditions and to restore β-catenin levels following stress exposure. As shown in Figure 4, treatment with Elissara at 200 µg/mL markedly increased β-catenin levels in the hair bulb region under basal conditions. Elissara significantly increased β-catenin levels to 182.9 ± 8.5% of the untreated control, corresponding to an 82.9% induction compared to the control group (*p* < 0.01).

Exposure to environmental stress significantly reduced β-catenin levels to 78.6 ± 4.8% of the control group (*p* < 0.05). However, treatment with Elissara counteracted this decrease, restoring β-catenin levels to 97.4 ± 6.6% of the control value. This effect corresponded to an 88% protective efficacy and was also statistically significant compared to the stress group (*p* < 0.05).

#### 3.3.2. Effect of Elissara on Ki67 Levels

**Figure 5:**
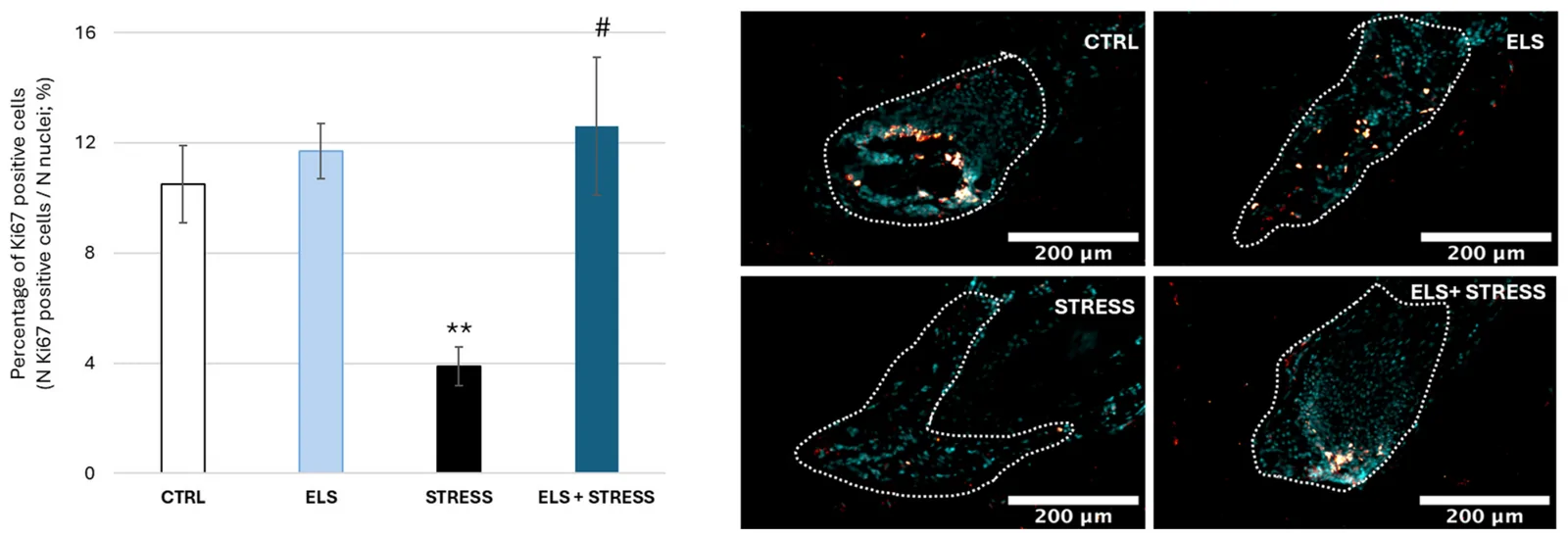
Effect of Elissara on Ki67-positive cells. Bar graphs represent the percentage of Ki67-positive cells relative to the total number of nuclei in the hair bulb region of human scalp explants after treatment with Elissara (ELS) at 200 µg/mL for three consecutive days, in the absence or presence of environmental stress. Data are presented as mean ± SD of 3 explants per condition. Asterisks indicate significant differences vs. CTRL: ** *p* < 0.01. Hash symbols indicate significant differences vs. STRESS: # *p* < 0.05. Representative images show in situ visualization of Ki67-positive signal in hair bulb region (a colour range: low levels in dark, high levels in bright colours) and superimposed on nuclear staining (DAPI, cyan). Images were acquired with a 10× objective. Regions of interest (ROI) are delineated with dotted lines. Scale bar = 200 µm.

Ki67 is a nuclear protein expressed during active phases of the cell cycle and is commonly used to assess proliferative activity in hair follicles [46,70]. UV radiation and particulate matter can induce oxidative stress and apoptotic responses in hair follicle cells, potentially reducing proliferative activity [71]. In this context, the percentage of Ki67-positive cells was evaluated in the hair bulb region as an indicator of follicular proliferative capacity. The results can be seen in Figure 5.

As shown, treatment with Elissara at 200 µg/mL under basal conditions slightly increased the percentage of Ki67-positive cells, reaching 11.7 ± 1.0% compared to 10.5 ± 1.4% in the untreated control group. However, this increase was not statistically significant (*p* = 0.31). In contrast, environmental stress markedly reduced the percentage of Ki67-positive cells to 3.9 ± 0.7%, representing a significant decrease compared to the untreated control group (*p* < 0.01). Treatment with Elissara counteracted the stress-induced reduction in Ki67-positive cells, increasing the percentage to 12.6 ± 2.5% (*p* < 0.05 vs. STRESS). This represented a 223.1% increase relative to the STRESS group, reflecting the low Ki67-positive cell percentage observed after PM_2.5_/UVA exposure. Importantly, the absolute Ki67 value in the ELS + STRESS group was restored to a range comparable to untreated control explants.

#### 3.3.3. Effect of Elissara on Bcl-2 Levels

Bcl-2 (B-cell lymphoma 2) plays a critical role in regulating cell survival within hair follicles. In hair follicles, Bcl-2 is highly expressed in the bulge stem cell compartment and dermal papilla cells, where it contributes to cell survival and protects against programmed cell death. Bcl-2 expression varies during the human hair cycle and has been implicated in follicular survival and hair-cycle transitions [41,42,72]. Also, UV radiation and particulate matter can trigger oxidative stress and apoptotic responses in hair follicle cells, contributing to premature follicular regression [11,73].

**Figure 6:**
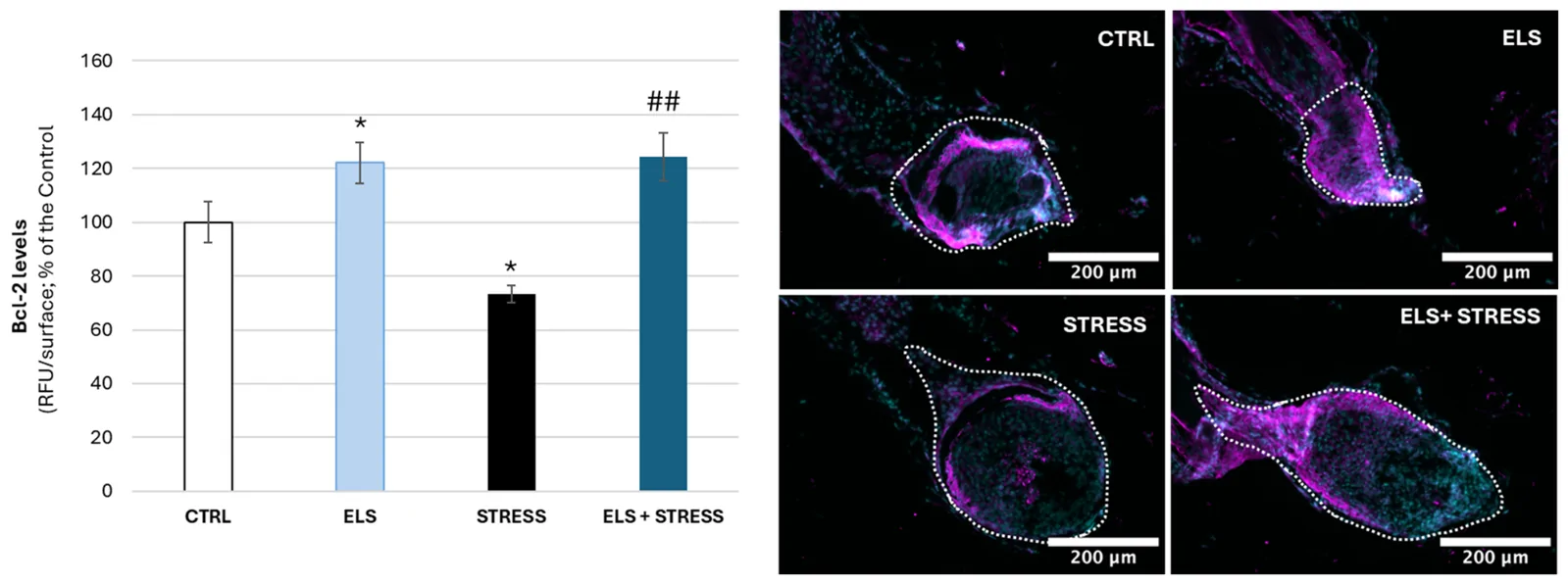
Effect of Elissara on Bcl-2 levels. Bar graphs represent Bcl-2 levels, normalized to untreated control (CTRL), in the hair bulb region of human scalp explants after treatment with Elissara (ELS) at 200 µg/mL for three consecutive days, in the absence or presence of environmental stress. Data are presented as mean ± SD of 3 explants per condition. Asterisks indicate significant differences vs. CTRL: * *p* < 0.05. Hash symbols indicate significant differences vs. STRESS: ## *p* < 0.01. Representative images show in situ visualization of Bcl-2 signal in follicular regions of scalp sections (purple), superimposed on nuclear detection (DAPI, cyan). Images were acquired with a 10× objective. Regions of interest (ROI) are delineated with dotted lines. Scale bar = 200 µm.

This study assessed the capacity of Elissara to increase the Bcl-2 levels under basal conditions and its ability to restore these levels following environmental stress conditions. As shown in Figure 6, under basal condition, treatment with Elissara significantly increased Bcl-2 levels to 122.1 ± 7.7% of the untreated control, corresponding to a 22% increase compared to the control group (*p* < 0.05). Environmental stress significantly reduced Bcl-2 levels to 73.4 ± 3.2% of the control group (*p* < 0.05). However, treatment with Elissara counteracted this stress-induced decrease, restoring Bcl-2 levels to 124.2 ± 8.8% of the control value (*p* < 0.01 vs. STRESS). This represented a 69.2% increase compared with the STRESS group, restoring the Bcl-2 signal to levels comparable to, or slightly above, those observed in untreated control explants. These findings suggest that Elissara may support anti-apoptotic or survival-associated signaling in hair follicles exposed to environmental stress.

#### 3.3.4. Effect of Elissara on IGF-1 Levels

IGF-1 (insulin-like growth factor 1) is a key growth factor that regulates cell proliferation, differentiation, and survival in hair follicles [44]. In hair follicles, IGF-1 is produced by dermal papilla cells and stimulates matrix keratinocyte proliferation, thereby promoting hair shaft elongation and maintenance of the anagen phase. IGF-1 also enhances cellular survival by counteracting apoptotic signals within the follicular microenvironment [43]. IGF-1 has also been reported to induce vascular endothelial growth factor (VEGF) expression in endothelial cells, suggesting a potential link with vascular support mechanisms relevant to the follicular microenvironment [74]. Exposure to UV rays directly decreases the synthesis of IGF-1 in the hair follicle outer root sheath [75].

**Figure 7:**
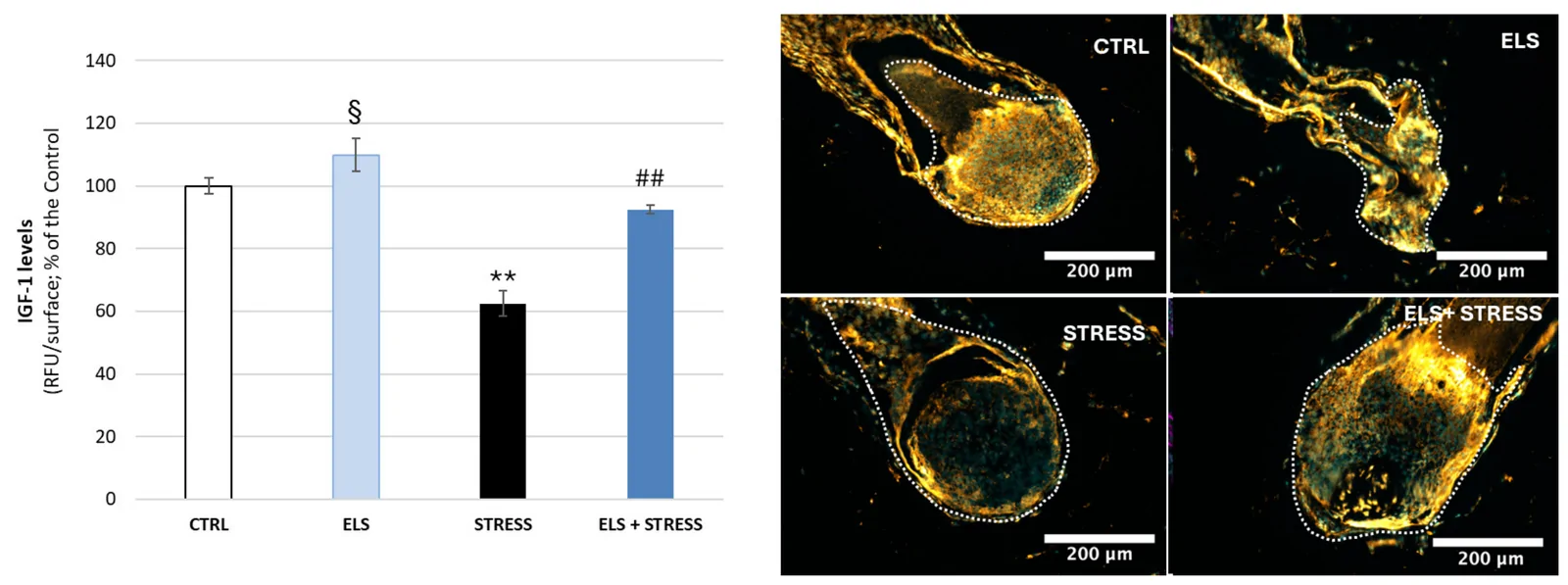
Effect of Elissara on IGF-1 levels. Bar graphs represent IGF-1 levels, normalized to untreated control (CTRL), in the hair bulb region of human scalp explants after treatment with Elissara (ELS) at 200 µg/mL for three consecutive days, in the absence or presence of environmental stress. Data are presented as mean ± SD of 3 explants per condition. Asterisks indicate significant differences vs. CTRL: ** *p* < 0.01. The symbol § indicates a statistical trend vs. CTRL: § *p* < 0.10. Hash symbols indicate significant differences vs. STRESS: ## *p* < 0.01. Representative images show in situ IGF-1 signal in the follicular region of scalp sections visualized as a colour intensity range (low levels in dark, high levels in bright colours) and superimposed to the nuclear detection (DAPI, cyan). Images were acquired with a 10× objective. Regions of interest (ROI) are delineated with a dotted line. Scale bar = 200 µm.

This study evaluated the ability of Elissara to increase IGF-1 levels under basal conditions and to restore IGF-1 expression following environmental stress. As shown in Figure 7, under basal conditions, scalp treatment with Elissara increased IGF-1 levels to 109.9 ± 5.1% of the untreated control, corresponding to a 10% increase, although this effect did not reach statistical significance (*p* = 0.06). Under environmental stress, IGF-1 levels were significantly reduced to 62.4 ± 4.1% of the control group (*p* < 0.01). Treatment with Elissara partially counteracted this stress-induced decrease, restoring IGF-1 immunofluorescence signal to 92.5 ± 1.3% of the control value (*p* < 0.01 vs. STRESS). This corresponded to a 48.2% increase compared with the STRESS group and an approximately 80% recovery of the stress-induced IGF-1 signal loss.

#### 3.3.5. Effect of Elissara on Collagen IV Levels

Collagen IV is a major structural component of the basement membrane that provides mechanical support and regulates cell–matrix interactions in hair follicles [47,48]. In hair follicles, it is primarily localized at the dermal–epidermal junction and around the outer root sheath, where it maintains structural integrity and supports follicular anchorage. UV-induced oxidative stress may compromise basement membrane integrity by promoting matrix-degrading activity, including MMP-mediated remodeling. In particular, MMP-2 and MMP-9 can degrade type IV collagen and other basement membrane components [76,77]. Therefore, disruption of type IV collagen after environmental stress may weaken the follicular basement membrane and impair follicular architecture and stability.

**Figure 8:**
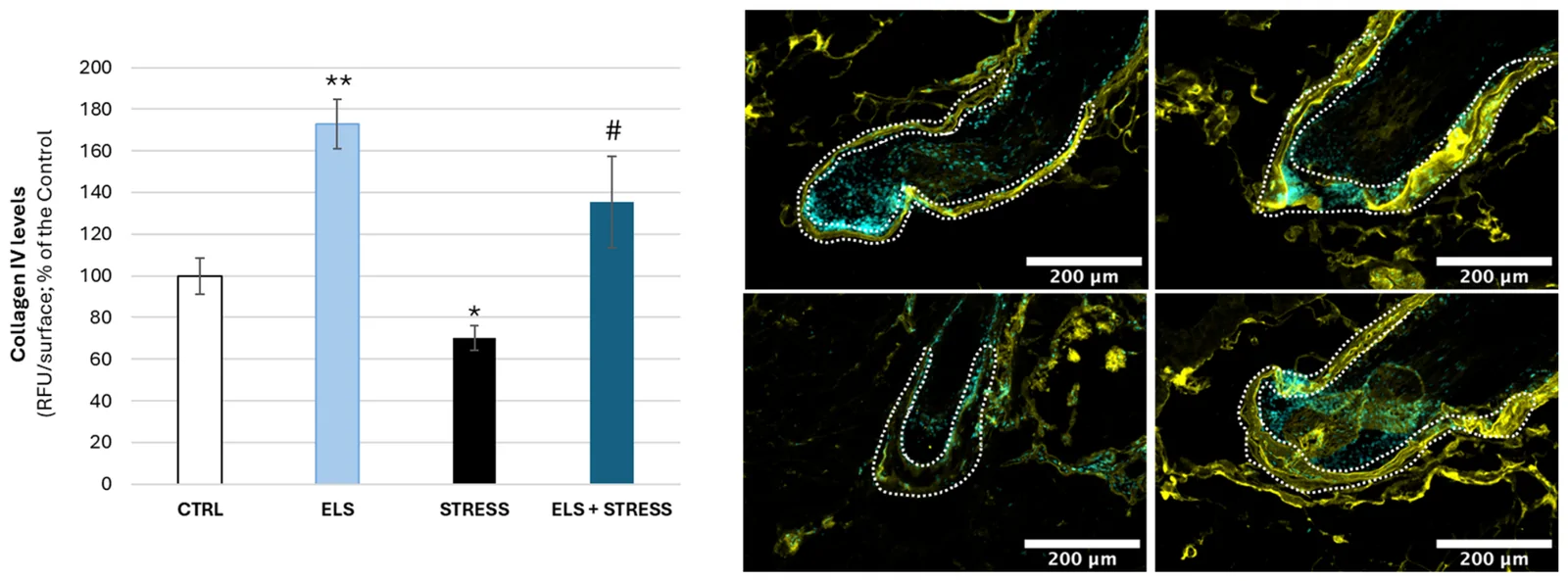
Effect of Elissara on collagen IV levels. Bar graphs represent collagen IV levels, normalized to untreated control (CTRL), in the hair bulb region of human scalp explants after treatment with Elissara (ELS) at 200 µg/mL for three consecutive days, in the absence or presence of environmental stress. Data are presented as mean ± SD of 3 explants per condition. Asterisks indicate significant differences vs. CTRL: * *p* < 0.05, ** *p* < 0.01. Hash symbols indicate significant differences vs. STRESS: # *p* < 0.05. Representative images show collagen IV signal in the follicular region of scalp sections (yellow), superimposed on nuclear staining (DAPI, cyan). Images were acquired with a 10× objective. Regions of interest (ROI) are delineated with a dotted line. Scale bar = 200 µm.

As shown in Figure 8, treatment with Elissara at 200 µg/mL under basal conditions increased collagen IV levels to 173.0 ± 11.9% of control (*p* < 0.01). Environmental stress reduced collagen IV levels to 70.0 ± 5.9% of control (*p* < 0.05), whereas Elissara restored levels to 135.4 ± 21.8% of control (*p* < 0.05 vs. STRESS), corresponding to a 93.4% increase compared with the STRESS group. This indicates recovery of the stress-induced reduction in collagen IV signal, with levels exceeding those observed in untreated control explants and suggesting preservation of basement membrane-associated matrix components.

#### 3.3.6. Effect of Elissara on Carbonylation Levels

Protein carbonylation is a marker of irreversible oxidative damage that occurs primarily when proteins are modified by reactive oxygen species [50,78]. In hair-related structures, increased protein carbonylation reflects oxidative damage to structural and functional proteins, which may compromise hair fibre integrity and follicular function [49,50]. UV-radiation and pollutants significantly increase protein carbonylation by generating ROS, thereby compromising follicular integrity and potentially promoting premature hair follicle regression [49,79].

**Figure 9:**
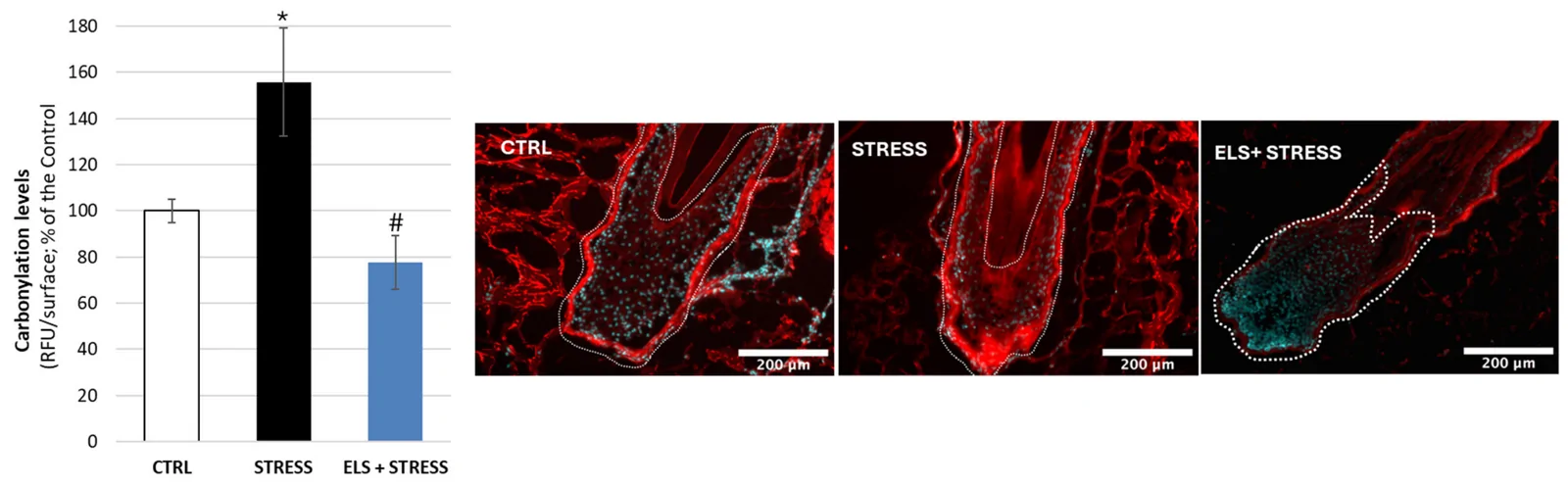
Effect of Elissara on carbonylation levels. Bar graphs represent carbonylation levels, normalized to untreated control (CTRL), in the hair bulb region of human scalp explants after treatment with Elissara (ELS) at 200 µg/mL under environmental stress conditions. Data are presented as mean ± SD of 3 explants per condition. Asterisks indicate significant differences vs. CTRL: * *p* < 0.05. Hash symbols indicate significant differences vs. STRESS: # *p* < 0.05. Representative images show carbonylation signal in the follicular region of scalp sections (red), superimposed on nuclear staining (DAPI, cyan). Images were acquired with a 10× objective. Regions of interest (ROI) are delineated with a dotted line. Scale bar = 200 µm.

As shown in Figure 9, exposure to environmental stress significantly increased protein carbonylation levels to 155.8 ± 23.4% of the untreated control group (*p* < 0.05), confirming the oxidative impact of PM_2.5_ and UV-A radiation on human scalp explants.

Treatment with Elissara markedly reduced the stress-induced increase in protein carbonylation, lowering the signal to 77.7 ± 11.6% of the untreated control group (*p* < 0.05 vs. STRESS). This indicates that Elissara prevented the stress-induced increase in oxidative protein damage, reducing carbonylation levels below those observed in untreated control explants. Overall, these results show that Elissara protects human scalp explants against oxidative protein damage induced by environmental stress, supporting its antioxidant protective effect at the follicular level.

## 4. Discussion

The present study provides a preclinical assessment of the follicle-related and stress-protective effects of Elissara, a botanical nutricosmetic ingredient, using complementary in silico, in vitro, and ex vivo approaches. Overall, the findings suggest that Elissara may influence several biological processes relevant to hair follicle homeostasis and resilience, including androgen-related pathway modulation, dermal papilla cell activity, Wnt/β-catenin-associated signaling, follicular cell survival, extracellular matrix integrity, and protection against oxidative damage. Rather than indicating a single dominant mechanism, these results support a multi-target profile in which Elissara may contribute to the preservation of follicular function under both basal and environmental stress conditions.

Hair loss and hair thinning are multifactorial processes involving the interaction of hormonal regulation, genetic predisposition, follicular cycling, inflammation, oxidative stress, extracellular matrix remodeling, and environmental exposure [1]. Although androgenetic alopecia is classically driven by androgen-dependent follicular miniaturization, mainly through the action of DHT in genetically susceptible follicles [6,7], increasing evidence indicates that exposome-related factors, including ultraviolet radiation, airborne pollutants, oxidative stress, and inflammation, can further impair follicular homeostasis and accelerate hair fiber weakening or shedding [8,13,14,15]. In this vein, oral botanical and nutraceutical ingredients with multi-target activity may be particularly relevant, as they can simultaneously address different pathways involved in hair growth and retention. This multi-target rationale has been applied in oral hair-support formulations combining anti-androgenic botanicals such as *Serenoa repens*, pumpkin seed oil, β-sitosterol, and *Pygeum africanum*; antioxidant and anti-inflammatory compounds such as curcumin and tocotrienols; adaptogens such as ashwagandha; and bioavailability-enhancing compounds such as piperine and capsaicin [80]. Other oral formulations combine L-cystine, *Serenoa repens*, *Cucurbita pepo*, *Pygeum africanum*, vitamins, and micronutrients in androgenetic alopecia and chronic telogen effluvium [81]. In addition, marine protein complex-based supplements combined with horsetail-derived silica, acerola/vitamin C, biotin, zinc, and other micronutrients have been clinically evaluated for promoting terminal hair growth and reducing shedding in women with self-perceived thinning hair [82]. In this context, Elissara, a standardized blend of *Lippia citriodora*, *Olea europaea, Sophora japonica*, and *Rosmarinus officinalis* extracts, providing a characteristic profile of bioactive compounds (verbascoside, oleuropein, hydroxytyrosol, quercetin, carnosic acid, and carnosol), aligns with a multi-target approach [23].

Previous studies with this ingredient demonstrated protective activity against pollution- and UV-induced oxidative stress, inflammatory responses, and AhR-related pathways in skin models [31,32]. In addition, oral supplementation with Elissara for 12 weeks improved scalp moisturization and reduced TEWL, sebum levels, and redness, while also improving self-perceived hair quality and brightness in women exposed to urban environments [33]. However, until now, no study had examined whether these benefits extend to direct modulation of follicular signaling pathways. The present study provides initial mechanistic evidence supporting this possibility, thereby extending the rationale for Elissara from a skin- and scalp-protective ingredient toward a nutricosmetic ingredient capable of supporting follicular health and resilience under environmental stress.

A key finding of this study is the coherence between computational predictions and experimental outcomes. Individual molecular docking showed that most of the representative Elissara bioactives exhibited moderate to favorable binding energies against the selected targets. The most favorable individual profiles were observed for the 5α-reductase isoforms, particularly SRD5A2, which is directly involved in the conversion of testosterone into DHT, a key mediator of follicular miniaturization in androgenetic alopecia. Most compounds, except hydroxytyrosol, showed binding energies ranging from −8.70 to −9.73 kcal/mol, approaching but not surpassing the reference inhibitors dutasteride (−11.93 kcal/mol), and finasteride (−11.30 kcal/mol), suggesting a potential modulatory activity at this critical enzyme. The predicted interaction patterns revealed that several compounds engaged key catalytic residues, including Glu197, Arg94, Trp53, Phe118, Phe223, and Leu224, which are known to be involved in substrate recognition and enzymatic function [55]. Among the botanical constituents, oleuropein exhibited the most favorable individual docking score against SRD5A2 (−9.73 kcal/mol), consistent with previous studies suggesting its potential as a 5α-reductase modulator and as an inducer of anagen-phase hair growth in telogen mouse skin [37]. In addition, simultaneous multi-ligand docking suggested that the active constituents could be accommodated within the SRD5A2 binding region in structurally plausible arrangements, although these results should be interpreted qualitatively and cannot be considered evidence of additive affinity or biological synergy.

At the cellular level, Elissara significantly reduced SRD5A2 levels in HFDPC, with the greatest reduction observed at 0.006%. Although finasteride produced the numerically strongest reduction, the difference between finasteride and Elissara at 0.006% did not reach statistical significance under the experimental conditions tested. This should not be interpreted as pharmacological equivalence, particularly because the present assay only quantified SRD5A2 protein levels. Nevertheless, the convergence between docking predictions and SRD5A2 protein modulation supports the hypothesis that Elissara may influence androgen-related follicular pathways implicated in follicular miniaturization. Further studies assessing enzymatic activity and DHT production would be needed to confirm this mechanism.

In addition to SRD5A2, the docking analyses also suggested potential interaction of several bioactives with SRD5A1. This observation is relevant when considering previous clinical findings showing reduced skin and scalp sebum levels after Elissara supplementation in subjects with oily skin type [33]. SRD5A1 is highly expressed in sebaceous glands and contributes to local DHT formation involved in sebaceous activity and sebum production [83]. The previously observed normalization of skin and scalp sebum may be partly due to modulation of androgen metabolism involving SRD5A1 in sebaceous structures and SRD5A2 in dermal papilla cells. Nevertheless, the SRD5A1 docking results should be interpreted with caution because this target was evaluated using an AlphaFold-predicted structure and SRD5A1 activity was not experimentally assessed in the present study. Further enzymatic assays are required to confirm whether Elissara or its individual constituents directly affect SRD5A1 activity.

Dermal papilla cells are essential regulators of epithelial–mesenchymal interactions, matrix keratinocyte proliferation, and hair cycle progression; thus, the stimulation of their proliferative capacity is considered a relevant indicator of hair growth-supporting potential [45]. Our in vitro proliferation assay showed that Elissara significantly increased BrdU incorporation in HFDPC at 0.002%, whereas the higher tested concentration (0.006%) did not induce proliferation. This non-concentration-dependent pattern is consistent with a hormetic, biphasic dose–response. This phenomenon has been described for numerous phytochemicals and botanical extracts [84], including several agents previously reported to stimulate hair growth and dermal papilla cell proliferation, where low concentrations promote proliferation while higher concentrations are neutral or inhibitory [85]. However, the relatively large variability observed in the 0.002% group highlights the need for broader dose–response studies.

The concentration-dependent proliferative response observed in HFDPC was also relevant when interpreting the ex vivo Ki67 and IGF-1 findings. The proliferation marker Ki67 and the growth factor IGF-1 exhibited a similar trend in scalp explants. Under basal conditions, Elissara produced modest, non-significant increases in Ki67-positive cells and IGF-1 levels (*p* = 0.31 and *p* = 0.06, respectively). In contrast, PM_2.5_/UVA exposure markedly reduced both parameters. Notably, Elissara counteracted these stress-induced reductions, restoring Ki67-positive cells to values comparable to or slightly above those observed in untreated control explants and partially restoring IGF-1 levels (80% of the loss induced by stress). Therefore, the recovery of both markers under PM_2.5_/UVA stress supports the idea that Elissara may help preserve a growth-supportive follicular microenvironment under environmental challenge.

The limited effects observed under basal conditions may reflect the fact that proliferative activity and IGF-1-associated signaling in healthy explants are already in a physiological range, leaving limited room for further stimulation. On the contrary, environmental exposure created a wider margin in which a protective effect could be detected. This interpretation is biologically coherent because Ki67 reflects proliferative activity [46], which can be suppressed by oxidative or UV-related stress [71], whereas IGF-1 is a dermal papilla-derived growth factor involved in matrix keratinocyte proliferation, hair shaft elongation, anagen maintenance, and potential vascular support mechanisms [43,44,74,75].

On the other hand, the modest basal effects on Ki67 and IGF-1 may also be related to the use of a single ex vivo concentration, which may not have matched the optimal range for stimulating these markers under unstressed conditions. This possibility is consistent with the HFDPC proliferation assay, where Elissara increased proliferation at 0.002% but not at 0.006%, suggesting a non-linear response that should be further explored in ex vivo dose–response studies.

Beyond proliferation and growth factor-related markers, β-catenin showed one of the clearest responses to Elissara treatment, supporting the role of this botanical blend in growth-associated follicular signaling. β-catenin is a core effector of the Wnt pathway and is essential for anagen initiation, follicular stem cell activation, and hair follicle morphogenesis [39,40,68,69]. In the present study, Elissara increased β-catenin levels under basal conditions and largely restored them following PM_2.5_/UVA-induced stress.

These findings are consistent with the in silico observations and are coherent with the activity previously described for some individual constituents of the blend. For example, oleuropein has been shown to increase β-catenin expression and nuclear accumulation in dermal papilla cells, together with the upregulation of key Wnt-related targets and follicular growth factors [37]. Other constituents, such as carnosic acid and verbascoside, may further contribute indirectly to the preservation of β-catenin-associated signaling by reducing oxidative, inflammatory, or androgen-related stress in the follicular microenvironment [35,86]. A similar pattern was observed for Bcl-2, an anti-apoptotic protein highly expressed in the bulge stem cell compartment and dermal papilla, where it protects follicular cells from programmed cell death. By inhibiting mitochondrial apoptotic pathways, Bcl-2 helps maintain follicular cell viability and supports the persistence of the anagen phase [42,72]. Altered Bcl-2 expression and apoptosis-related signaling have been described in alopecia areata and androgenetic alopecia, supporting the relevance of survival pathways in hair loss-associated conditions [87,88]. In addition, UV radiation and particulate matter can trigger oxidative stress and apoptotic responses in hair follicle cells, contributing to premature follicle regression [11,73]. In this study, Elissara increased Bcl-2 levels under basal conditions and restored Bcl-2 signal after PM_2.5_/UVA-induced stress. These findings suggest that Elissara may reinforce anti-apoptotic defenses within the follicular microenvironment, particularly under oxidative or inflammatory stress conditions. These results are consistent with the antioxidant and anti-inflammatory properties of its active compounds, including hydroxytyrosol, oleuropein, verbascoside, quercetin, carnosic acid, and carnosol [26,30,89,90]. They are also in agreement with previous studies reporting hair-protective effects of several of these bioactive compounds. For instance, verbascoside has been shown to prevent testosterone-induced apoptosis in human dermal papilla cells, while also promoting dermal papilla cell proliferation and reducing the release of inflammatory mediators [35]. Hydroxytyrosol has been reported to protect dermal papilla cells under oxidative stress by reducing intracellular ROS, apoptotic markers, and inflammation, while enhancing cell survival through autophagy regulation [34]. In addition, quercetin was recently shown to rescue DHT-treated human dermal papilla cells by preserving mitochondrial function, reducing oxidative damage, and suppressing apoptosis/autophagy through SHP2/AKT signaling [38]. Altogether, these findings provide a mechanistic context for the restoration of Bcl-2 levels observed in the present study, suggesting that Elissara may support follicular cell survival through the combined anti-apoptotic, antioxidant, and anti-inflammatory actions of its polyphenolic constituents.

The preservation and basal increase of collagen IV in scalp explants provides further mechanistic understanding into the follicular protective activity of Elissara. Collagen IV is a major structural component of the follicular basement membrane, essential for the integrity of the epithelial–mesenchymal interface and the maintenance of the hair follicle stem cell niche [47,48]. Collagen IV and its remodeling are functionally relevant to hair follicle cycling, and type IV collagenase activity has been associated with regulation of hair-cycle-related mediators such as VEGF, IGF-1, and TGF-β [91]. UV radiation and particulate matter can induce extracellular matrix (ECM) remodelling through the activation of matrix metalloproteinases (MMPs). In this sense, MMP-2 and MMP-9 are particularly relevant because they act as collagenases/gelatinases capable of degrading type IV collagen and other basement membrane components [77]. Since MMP-2 and MMP-9 are involved in hair follicle matrix remodeling during the hair cycle, their stress-induced dysregulation may compromise collagen IV preservation, disrupt epithelial–mesenchymal interactions, and weaken the ECM scaffold supporting follicular architecture [47,48,91].

The restoration of collagen IV observed after environmental exposure by Elissara in this study is consistent with the documented ability of some of its bioactives to reduce MMP activity. For instance, oleuropein and hydroxytyrosol have been shown to moderately inhibit collagenase and elastase activity in skin fibroblasts [92], and a combination of these two compounds with verbascoside has recently been reported to reduce MMP-1 and MMP-9 expression in UV-irradiated skin cells through coordinated MAPK/NF-κB inhibition and Nrf2 activation [93]. Quercetin has similarly been reported to suppress UV-induced MMP activity in human skin tissues [94]. Beyond MMP inhibition, the basal increase in collagen IV observed in unstressed explants suggests that Elissara may also favour extracellular matrix maintenance or synthesis. In support of this interpretation, olive-derived polyphenols, including oleuropein and hydroxytyrosol, have been associated with stimulation of collagen synthesis in fibroblast models [92].

These findings may also provide a plausible mechanistic context for the improvements in skin firmness and wrinkle appearance previously reported in subjects supplemented with Elissara [32,33], since the same collagen-protective pathways operating at the follicular basement membrane level may contribute more broadly to dermal collagen homeostasis. However, this connection remains hypothetical, and dedicated studies specifically designed to assess dermal collagen synthesis, MMP activity, and basement membrane remodelling would be needed to confirm this mechanism.

The prevention of environmental stress-induced protein carbonylation represents the most direct antioxidant readout observed in the present study. Protein carbonylation is an irreversible marker of oxidative protein damage that has been linked to hair shaft deterioration and impaired follicular function [49,50]. The ability of Elissara to prevent the stress-induced increase in carbonylation, reducing the signal below untreated control levels, is particularly consistent with previous evidence obtained with the same botanical formulation, formerly developed as Zeropollution^®^. In previous preclinical studies, this ingredient reduced intracellular ROS generation in keratinocytes exposed to UV radiation and particulate matter, and decreased malondialdehyde (MDA), a marker of lipid peroxidation, in skin explants exposed to pollution-related stress [31]. In addition, previous clinical studies showed that oral supplementation with this botanical blend improved antioxidant status, as reflected by increased FRAP/FRAS values in skin and saliva, respectively [32,33]. Therefore, the present reduction in protein carbonylation extends the antioxidant activity previously demonstrated in follicular regions of the scalp explant model.

In summary, the present study suggests that Elissara may exert its biological activity through three complementary mechanisms: modulation of androgen-related pathways, preservation of hair follicle growth and survival signaling, and protection of the follicular microenvironment against exposome-induced oxidative stress and extracellular matrix damage. Specifically, Elissara reduced SRD5A2 protein levels in dermal papilla cells, preserved β-catenin- and Bcl-2-associated signals, maintained collagen IV within the follicular niche, and reduced oxidative damage under environmental stress conditions. Taken together, these findings provide new insights into the mechanisms underlying the effects of Elissara, expanding its established role from a purely skin and scalp-protective ingredient to a promising nutricosmetic agent capable of actively supporting hair follicle homeostasis and stress resilience.

Because Elissara is a pre-established, standardized botanical blend, the present study was not designed as a formulation-optimization or synergy-testing study. Rather, it aimed to determine whether this defined botanical combination could influence biological pathways relevant to hair follicle homeostasis. Therefore, isolated compounds and pairwise combinations were not tested, and the contribution of individual constituents or potential additive or synergistic interactions remains to be determined in future studies.

Nevertheless, some limitations should be considered. First, the molecular docking and simultaneous multi-ligand docking analyses should be interpreted as exploratory and hypothesis-generating, rather than direct evidence of biological activity or biological synergy. In addition, docking against the SRD5A1 isoform was performed using an AlphaFold-predicted structure, and its potential modulation was not confirmed by functional assays.

Second, the SRD5A2 study in HFDPC did not directly assess enzymatic activity or dihydrotestosterone (DHT) production. Future studies should therefore include specific enzymatic assays and DHT quantification to better characterize the androgen-related effects of Elissara against both 5α-reductase isoforms.

Furthermore, the in vitro experiments were conducted using a limited range of concentrations, and optimal dose–response relationships remain to be established. In addition, the ex vivo study was conducted at a single concentration selected based on prior skin explant studies. Whether this represents the optimal effective concentration for follicular biomarkers in scalp tissue remains to be determined, and dose–response studies in this model would be needed. Also, the use of scalp explants from a single donor further limits the generalizability of the findings, as inter-individual variability was not captured related to age, sex, hormonal status, or hair-loss phenotype. However, the coherent results observed across the evaluated biomarkers support the robustness of the experimental findings and provide a rationale for further investigation in larger, donor-diverse studies.

Finally, although this study provides mechanistic evidence through relevant biomarker modulation, it does not include direct evaluation of clinical hair growth outcomes. Therefore, clinical studies are warranted to determine whether oral supplementation with Elissara translates into measurable benefits in individuals experiencing hair thinning or early-stage hair loss, including improvements in the anagen–telogen ratio, hair density, and hair shedding.

## 5. Conclusions

The study provides preclinical evidence that Elissara^®^ may influence several biological pathways relevant to hair follicle homeostasis and resilience, including androgen-related pathway modulation, dermal papilla cell activity, growth-associated signaling, follicular cell survival, extracellular matrix integrity, and protection against oxidative damage. Rather than acting through a single mechanism, this botanical blend appears to exert a multi-target profile that extends its previously described scalp and skin benefits toward the preservation of hair follicle function under basal and acute environmental stress conditions. These findings highlight the potential of Elissara^®^ as a promising nutricosmetic ingredient for maintaining follicular homeostasis and supporting scalp and hair health. Further clinical evaluation would be needed to determine whether these preclinical findings translate into measurable benefits in individuals experiencing hair thinning or increased hair shedding.

## Figures and Tables

**Table 1:** Receptor structures and grid box parameters used for molecular docking.

Target	Structure Used	Grid Center X, Y, Z Å	Grid Dimensions X × Y × Z Å
SRD5A2	7BW1, X-ray, 3.0 Å	−28.826, 17.949, 28.959	21 × 21 × 21
SRD5A1	AF-P18405-F1-v6, AlphaFold model	−13.266, 2.112, 3.680	23.9 × 23.9 × 28.8
β-Catenin	1JDH, X-ray, 2.2 Å	−5.671, 8.506, 37.775	30.3 × 25.0 × 33.6
BCL-2	4MAN, X-ray, 2.0 Å	−15.634, 15.175, 5.010	20 × 28 × 28
IGF-1R	2OJ9, X-ray, 2.6 Å	9.442, 1.491, 20.205	14.6 × 20.2 × 18.9

Grid centers and dimensions are expressed in Å. SRD5A1 was modeled using AlphaFold due to the absence of a suitable experimental structure.

**Table 2:** Experimental design of the ex vivo scalp explant study.

Batch	Description	Days 1–3	Day 4	No. of Explants	Sampling Time
CTRL	Untreated control	Culture medium only	No stress	3	Day 5
ELS	Elissara treatment	Elissara at 200 µg/mL	No stress	3	Day 5
STRESS	Stress control	Culture medium only	PM_2.5_ + UVA	3	Day 5
ELS + STRESS	Elissara + stress	Elissara at 200 µg/mL	PM_2.5_ + UVA	3	Day 5

CTRL, untreated control; ELS, Elissara; STRESS, PM_2.5_-like particles plus UVA exposure. Elissara was applied in the culture media once daily during Days 1–3. Samples were collected on Day 5, 24 h after stress exposure or at the corresponding time point under non-stressed conditions.

**Table 3:** AutoDock Vina (version 1.1.2) docking scores: binding energy (kcal/mol) from molecular docking simulations.

	SRD5A2	SRD5A1	β-Catenin	BCL-2	IGF-1R
**Reference compounds**					
Dutasteride	−11.93	−10.45	—	—	—
Finasteride	−11.30	−7.20	—	—	—
Sitosterol	−11.13	−8.77	—	—	—
iCRT14	—	—	−6.80	—	—
Venetoclax	—	—	—	−10.60	—
Benzimidazole	—	—	—	—	−7.53
**Botanical compounds**					
Verbascoside	−9.47	−10.37	−6.60	−8.20	−6.80
Quercetin	−9.10	−9.17	−6.50	−6.60	−8.00
Hydroxytyrosol	−5.80	−5.97	−4.57	−5.20	−5.67
Oleuropein	−9.73	−9.63	−6.57	−7.13	−6.17
Carnosic acid	−8.70	−8.47	−6.00	−6.63	−6.57
Carnosol	−9.20	−8.83	−6.10	−6.97	−6.33
**Simultaneous** **Multi-ligand docking**	−15.65	−7.05	−15.73	−16.44	−16.43

Values are expressed as kcal/mol. Simultaneous multi-ligand docking values represent exploratory global outputs obtained when all six botanical compounds were docked simultaneously against a fixed receptor; they should be interpreted solely as hypotheses of spatial compatibility and not as evidence of stable co-occupation, experimental binding affinity, or biological synergy. — indicates compound not tested against this target.

## Data Availability

The original contributions presented in this study are included in the article/Supplementary Material. Further inquiries can be directed to the corresponding authors.

## Supplementary Materials

The following supporting information can be downloaded at: https://www.mdpi.com/article/10.3390/biom16081207/s1. Figure S1: Cytotoxicity assessment of Elissara^®^ in human follicle dermal papilla cells (HFDPC) after 24 h of treatment.

## References

[1] Sowińska T., Łabuda M., Słojewska A., Kotkowiak A., Mentel O., Bogucka A., Szema A., Królikowska K., Sikora J., Knychalska K. (2025). Hair Loss: Pathogenesis and Prevention: A Literature Review. Qual. Sport.

[2] Otberg N., Finner A.M., Shapiro J. (2007). Androgenetic Alopecia. Endocrinol. Metab. Clin. N. Am..

[3] Alonso L., Fuchs E. (2006). The Hair Cycle. J. Cell Sci..

[4] Natarelli N., Gahoonia N., Sivamani R.K. (2023). Integrative and Mechanistic Approach to the Hair Growth Cycle and Hair Loss. J. Clin. Med..

[5] Gupta A.K., Economopoulos V., Mann A., Wang T., Mirmirani P. (2025). Menopause and Hair Loss in Women: Exploring the Hormonal Transition. Maturitas.

[6] Sawaya M.E., Price V.H. (1997). Different Levels of 5alpha-Reductase Type I and II, Aromatase, and Androgen Receptor in Hair Follicles of Women and Men with Androgenetic Alopecia. J. Investig. Dermatol..

[7] Hatanaka T., Lulic Z., Mefo T., Booth C., Harrison E., Ong G. (2022). Change in Hair Growth-Related Gene Expression Profile in Human Isolated Hair Follicles Induced by 5-Alpha Reductase Inhibitors—Dutasteride and Finasteride—In the Presence of Testosterone. Singap. Med. J..

[8] Cedirian S., Prudkin L., Piraccini B.M., Santamaria J., Piquero-Casals J., Saceda-Corralo D. (2025). The Exposome Impact on Hair Health: Etiology, Pathogenesis and Clinical Features—Part I. An. Bras. Dermatol..

[9] Samra T., Lin R.R., Maderal A.D. (2024). The Effects of Environmental Pollutants and Exposures on Hair Follicle Pathophysiology. Ski. Appendage Disord..

[10] Trüeb R.M., Henry J.P., Davis M.G., Schwartz J.R. (2018). Scalp Condition Impacts Hair Growth and Retention via Oxidative Stress. Int. J. Trichol..

[11] Jun M.S., Kwack M.H., Kim M.K., Kim J.C., Sung Y.K. (2020). Particulate Matters Induce Apoptosis in Human Hair Follicular Keratinocytes. Ann. Dermatol..

[12] Boo Y.C. (2019). Can Plant Phenolic Compounds Protect the Skin from Airborne Particulate Matter?. Antioxidants.

[13] Trüeb R.M. (2021). Oxidative Stress and Its Impact on Skin, Scalp and Hair. Int. J. Cosmet. Sci..

[14] Du F., Li J., Zhang S., Zeng X., Nie J., Li Z. (2024). Oxidative Stress in Hair Follicle Development and Hair Growth: Signalling Pathways, Intervening Mechanisms and Potential of Natural Antioxidants. J. Cell. Mol. Med..

[15] Trüeb R.M. (2015). The Impact of Oxidative Stress on Hair. Int. J. Cosmet. Sci..

[16] Ma Y., Sun Z., Li Y.-M., Xu H. (2023). Oxidative Stress and Alopecia Areata. Front. Med..

[17] Prie B., Iosif L., Tivig I., Stoian I., Giurcaneanu C. (2016). Oxidative Stress in Androgenetic Alopecia. J. Med. Life.

[18] Ramos P.M., Brianezi G., Martins A.C.P., da Silva M.G., Marques M.E.A., Miot H.A. (2017). Aryl Hydrocarbon Receptor Overexpression in Miniaturized Follicles in Female Pattern Hair Loss. An. Bras. Dermatol..

[19] Evron E., Juhasz M., Babadjouni A., Mesinkovska N.A. (2020). Natural Hair Supplement: Friend or Foe? Saw Palmetto, a Systematic Review in Alopecia. Ski. Appendage Disord..

[20] Kwon O.S., Han J.H., Yoo H.G., Chung J.H., Cho K.H., Eun H.C., Kim K.H. (2007). Human Hair Growth Enhancement in Vitro by Green Tea Epigallocatechin-3-Gallate (EGCG). Phytomedicine.

[21] Choi J.Y., Boo M.Y., Boo Y.C. (2024). Can Plant Extracts Help Prevent Hair Loss or Promote Hair Growth? A Review Comparing Their Therapeutic Efficacies, Phytochemical Components, and Modulatory Targets. Molecules.

[22] Cho Y.H., Lee S.Y., Jeong D.W., Choi E.J., Kim Y.J., Lee J.G., Yi Y.H., Cha H.S. (2014). Effect of Pumpkin Seed Oil on Hair Growth in Men with Androgenetic Alopecia: A Randomized, Double-Blind, Placebo-Controlled Trial. Evid. Based Complement. Altern. Med..

[23] Caturla Cernuda N., Peral Clement A. (2019). Composición De Extractos Vegetales Con Flavonoides Para Paliar Los Múltiples Efectos De La Contaminación Del Aire Sobre La Piel. International Patent Application.

[24] Jin X., Su R., Li R., Song L., Chen M., Cheng L., Li Z. (2016). Amelioration of Particulate Matter-Induced Oxidative Damage by Vitamin c and Quercetin in Human Bronchial Epithelial Cells. Chemosphere.

[25] Bertelli M., Kiani A.K., Paolacci S., Manara E., Kurti D., Dhuli K., Bushati V., Miertus J., Pangallo D., Baglivo M. (2020). Hydroxytyrosol: A Natural Compound with Promising Pharmacological Activities. J. Biotechnol..

[26] Pojero F., Aiello A., Gervasi F., Caruso C., Ligotti M.E., Calabrò A., Procopio A., Candore G., Accardi G., Allegra M. (2022). Effects of Oleuropein and Hydroxytyrosol on Inflammatory Mediators: Consequences on Inflammaging. Int. J. Mol. Sci..

[27] Nieto G., Ros G., Castillo J. (2018). Antioxidant and Antimicrobial Properties of Rosemary (*Rosmarinus officinalis*, L.): A Review. Medicines.

[28] Korkina L.G., Mikhal’chik E., Suprun M.V., Pastore S., Dal Toso R. (2007). Molecular Mechanisms Underlying Wound Healing and Anti-Inflammatory Properties of Naturally Occurring Biotechnologically Produced Phenylpropanoid Glycosides. Cell. Mol. Biol. Noisy--Gd. Fr..

[29] Espinosa-González A.M., García-Bores A.M., Benítez-Flores J.d.C., Sandoval-Pérez C.E., González-Valle M.d.R., Céspedes C.L., Avila-Acevedo J.G. (2016). Photoprotective effect of verbascoside from Buddleja cordata in SKH-1 mice exposed to acute and chronic UV-B radiation. Bol. Latinoam. Caribe Plantas Med. Aromáticas.

[30] Chiou W.F., Lin L.C., Chen C.F. (2004). Acteoside Protects Endothelial Cells against Free Radical-Induced Oxidative Stress. J. Pharm. Pharmacol..

[31] Peno-Mazzarino L., Radionov N., Merino M., González S., Mullor J.L., Jones J., Caturla N. (2024). Protective Potential of a Botanical-Based Supplement Ingredient against the Impact of Environmental Pollution on Cutaneous and Cardiopulmonary Systems: Preclinical Study. Curr. Issues Mol. Biol..

[32] Nobile V., Schiano I., Peral A., Giardina S., Spartà E., Caturla N. (2021). Antioxidant and Reduced Skin-Ageing Effects of a Polyphenol-Enriched Dietary Supplement in Response to Air Pollution: A Randomized, Double-Blind, Placebo-Controlled Study. Food Nutr. Res..

[33] Nobile V., Cestone E., Ghirlanda S., Poggi A., Navarro P., García A., Jones J., Caturla N. (2024). Skin and Scalp Health Benefits of a Specific Botanical Extract Blend: Results from a Double-Blind Placebo-Controlled Study in Urban Outdoor Workers. Cosmetics.

[34] Chen Q., Sun T., Wang J., Jia J., Yi Y.-H., Chen Y.-X., Miao Y., Hu Z.-Q. (2019). Hydroxytyrosol Prevents Dermal Papilla Cells Inflammation under Oxidative Stress by Inducing Autophagy. J. Biochem. Mol. Toxicol..

[35] Wisuitiprot V., Ingkaninan K., Chakkavittumrong P., Wisuitiprot W., Neungchamnong N., Chantakul R., Waranuch N. (2022). Effects of *Acanthus ebracteatus* Vahl. Extract and Verbascoside on Human Dermal Papilla and Murine Macrophage. Sci. Rep..

[36] Singh P., Kushwaha P., Ahmad M., Husain A. (2025). Therapeutic Potential of Carnosic Acid in Alopecia: A Mechanistic Perspective. Planta Med..

[37] Tong T., Kim N., Park T. (2015). Topical Application of Oleuropein Induces Anagen Hair Growth in Telogen Mouse Skin. PLoS ONE.

[38] Guo T., Li W., Zheng W., Lin Y., Wen S. (2025). Quercetin Rescues Dihydrotestosterone-Treated Human Dermal Papilla Cells via SHP2/AKT Signaling to Suppress Autophagy and Apoptosis. Naunyn. Schmiedebergs Arch. Pharmacol..

[39] Shin D.W. (2022). The Molecular Mechanism of Natural Products Activating Wnt/β-Catenin Signaling Pathway for Improving Hair Loss. Life.

[40] Huelsken J., Vogel R., Erdmann B., Cotsarelis G., Birchmeier W. (2001). β-Catenin Controls Hair Follicle Morphogenesis and Stem Cell Differentiation in the Skin. Cell.

[41] Soma T., Hibino T. (2004). Dominant Bcl-2 Expression during Telogen–Anagen Transition Phase in Human Hair. J. Dermatol. Sci..

[42] Geueke A., Mantellato G., Kuester F., Schettina P., Nelles M., Seeger J.M., Kashkar H., Niemann C. (2021). The Anti-apoptotic Bcl-2 Protein Regulates Hair Follicle Stem Cell Function. EMBO Rep..

[43] Ahn S.-Y., Pi L.-Q., Hwang S.T., Lee W.-S. (2012). Effect of IGF-I on Hair Growth Is Related to the Anti-Apoptotic Effect of IGF-I and Up-Regulation of PDGF-A and PDGF-B. Ann. Dermatol..

[44] Weger N., Schlake T. (2005). Igf-I Signalling Controls the Hair Growth Cycle and the Differentiation of Hair Shafts. J. Investig. Dermatol..

[45] Chi W., Enshell-Seijffers D., Morgan B.A. (2010). De Novo Production of Dermal Papilla Cells during the Anagen Phase of the Hair Cycle. J. Investig. Dermatol..

[46] Sun X., Kaufman P.D. (2018). Ki-67: More than a Proliferation Marker. Chromosoma.

[47] Messenger A.G., Elliott K., Temple A., Randall V.A. (1991). Expression of Basement Membrane Proteins and Interstitial Collagens in Dermal Papillae of Human Hair Follicles. J. Investig. Dermatol..

[48] Jahoda C.A., Mauger A., Bard S., Sengel P. (1992). Changes in Fibronectin, Laminin and Type IV Collagen Distribution Relate to Basement Membrane Restructuring during the Rat Vibrissa Follicle Hair Growth Cycle. J. Anat..

[49] Grosjacques C., Babiel S., Hodes J., Bobier A., Cavagnino A., Baraibar M. (2025). Carbonylation of Hair Proteins: A Robust Biomarker of Molecular and Structural Oxidative Damage in Hair Fibres. Int. J. Cosmet. Sci..

[50] Fedorova M., Bollineni R.C., Hoffmann R. (2014). Protein Carbonylation as a Major Hallmark of Oxidative Damage: Update of Analytical Strategies. Mass. Spectrom. Rev..

[51] Souers A.J., Leverson J.D., Boghaert E.R., Ackler S.L., Catron N.D., Chen J., Dayton B.D., Ding H., Enschede S.H., Fairbrother W.J. (2013). ABT-199, a Potent and Selective BCL-2 Inhibitor, Achieves Antitumor Activity While Sparing Platelets. Nat. Med..

[52] Gonsalves F.C., Klein K., Carson B.B., Katz S., Ekas L.A., Evans S., Nagourney R., Cardozo T., Brown A.M.C., DasGupta R. (2011). An RNAi-Based Chemical Genetic Screen Identifies Three Small-Molecule Inhibitors of the Wnt/Wingless Signaling Pathway. Proc. Natl. Acad. Sci. USA.

[53] Graham T.A., Ferkey D.M., Mao F., Kimelman D., Xu W. (2001). Tcf4 Can Specifically Recognize Beta-Catenin Using Alternative Conformations. Nat. Struct. Mol. Biol..

[54] Velaparthi U., Wittman M., Liu P., Stoffan K., Zimmermann K., Sang X., Carboni J., Li A., Attar R., Gottardis M. (2007). Discovery and Initial SAR of 3-(1*H*-Benzo[*d*]Imidazol-2-Yl)Pyridin-2(1*H*)-Ones as Inhibitors of Insulin-like Growth Factor 1-Receptor (IGF-1R). Bioorg. Med. Chem. Lett..

[55] Xiao Q., Wang L., Supekar S., Shen T., Liu H., Ye F., Huang J., Fan H., Wei Z., Zhang C. (2020). Structure of Human Steroid 5α-Reductase 2 with the Anti-Androgen Drug Finasteride. Nat. Commun..

[56] Buț M.-G., Tero-Vescan A., Pușcaș A., Jîtcă G., Marc G. (2024). Exploring the Inhibitory Potential of Phytosterols β-Sitosterol, Stigmasterol, and Campesterol on 5-Alpha Reductase Activity in the Human Prostate: An In Vitro and In Silico Approach. Plants.

[57] Stelzer G., Rosen N., Plaschkes I., Zimmerman S., Twik M., Fishilevich S., Stein T.I., Nudel R., Lieder I., Mazor Y. (2016). The GeneCards Suite: From Gene Data Mining to Disease Genome Sequence Analyses. Curr. Protoc. Bioinform..

[58] Uhlén M., Fagerberg L., Hallström B.M., Lindskog C., Oksvold P., Mardinoglu A., Sivertsson Å., Kampf C., Sjöstedt E., Asplund A. (2015). Tissue-Based Map of the Human Proteome. Science.

[59] Szklarczyk D., Kirsch R., Koutrouli M., Nastou K., Mehryary F., Hachilif R., Gable A.L., Fang T., Doncheva N.T., Pyysalo S. (2023). The STRING Database in 2023: Protein–Protein Association Networks and Functional Enrichment Analyses for Any Sequenced Genome of Interest. Nucleic Acids Res..

[60] Kuhn M., Szklarczyk D., Pletscher-Frankild S., Blicher T.H., von Mering C., Jensen L.J., Bork P. (2014). STITCH 4: Integration of Protein–Chemical Interactions with User Data. Nucleic Acids Res..

[61] Ragueneau E., Gong C., Sinquin P., Sevilla C., Beavers D., Grentner A., Griss J., Hogue G.F.J., Li N.T., Matthews L. (2026). The Reactome Knowledgebase 2026. Nucleic Acids Res..

[62] Volkamer A., Griewel A., Grombacher T., Rarey M. (2010). Analyzing the Topology of Active Sites: On the Prediction of Pockets and Subpockets. J. Chem. Inf. Model..

[63] (2009). Biological Evaluation of Medical Devices Part 5: Tests for in Vitro Cytotoxicity.

[64] Hawker J.R. (2003). Chemiluminescence-Based BrdU ELISA to Measure DNA Synthesis. J. Immunol. Methods.

[65] Baraibar M.A., Ladouce R., Friguet B. (2013). Proteomic Quantification and Identification of Carbonylated Proteins upon Oxidative Stress and during Cellular Aging. J. Proteom..

[66] Schneider C.A., Rasband W.S., Eliceiri K.W. (2012). NIH Image to ImageJ: 25 Years of Image Analysis. Nat. Methods.

[67] Escamilla-Cruz M., Magaña M., Escandón-Perez S., Bello-Chavolla O.Y. (2023). Use of 5-Alpha Reductase Inhibitors in Dermatology: A Narrative Review. Dermatol. Ther..

[68] Ryu Y.C., Kim Y.-R., Park J., Choi S., Kim G.-U., Kim E., Hwang Y., Kim H., Bak S.S., Lee J.E. (2022). Wnt/β-Catenin Signaling Activator Restores Hair Regeneration Suppressed by Diabetes Mellitus. BMB Rep..

[69] Mehta A., Motavaf M., Raza D., McLure A.J., Osei-Opare K.D., Bordone L.A., Gru A.A. (2025). Revolutionary Approaches to Hair Regrowth: Follicle Neogenesis, Wnt/ß-Catenin Signaling, and Emerging Therapies. Cells.

[70] Ravali B.D., Thatavarthi N.B.L., Raju D.V.P.K., Rao D.S. (2025). Role of Ki67 Proliferative Index in Various Alopecia—A Pilot Study. Indian. J. Postgrad. Dermatol..

[71] Lu Z., Fischer T.W., Hasse S., Sugawara K., Kamenisch Y., Krengel S., Funk W., Berneburg M., Paus R. (2009). Profiling the Response of Human Hair Follicles to Ultraviolet Radiation. J. Investig. Dermatol..

[72] Lindner G., Botchkarev V.A., Botchkareva N.V., Ling G., van der Veen C., Paus R. (1997). Analysis of Apoptosis during Hair Follicle Regression (Catagen). Am. J. Pathol..

[73] Müller-Röver S., Rossiter H., Paus R., Handjiski B., Peters E.M.J., Murphy J.-E., Mecklenburg L., Kupper T.S. (2000). Overexpression of Bcl-2 Protects from Ultraviolet B-Induced Apoptosis but Promotes Hair Follicle Regression and Chemotherapy-Induced Alopecia. Am. J. Pathol..

[74] Beckert S., Farrahi F., Perveen Ghani Q., Aslam R., Scheuenstuhl H., Coerper S., Königsrainer A., Hunt T.K., Hussain M.Z. (2006). IGF-I-Induced VEGF Expression in HUVEC Involves Phosphorylation and Inhibition of Poly(ADP-Ribose)Polymerase. Biochem. Biophys. Res. Commun..

[75] Gherardini J., Wegner J., Chéret J., Ghatak S., Lehmann J., Alam M., Jimenez F., Funk W., Böhm M., Botchkareva N.V. (2019). Transepidermal UV Radiation of Scalp Skin Ex Vivo Induces Hair Follicle Damage That Is Alleviated by the Topical Treatment with Caffeine. Int. J. Cosmet. Sci..

[76] Roach D.M., Fitridge R.A., Laws P.E., Millard S.H., Varelias A., Cowled P.A. (2002). Up-Regulation of MMP-2 and MMP-9 Leads to Degradation of Type IV Collagen during Skeletal Muscle Reperfusion Injury; Protection by the MMP Inhibitor, Doxycycline. Eur. J. Vasc. Endovasc. Surg..

[77] Feng C., Chen X., Yin X., Jiang Y., Zhao C. (2024). Matrix Metalloproteinases on Skin Photoaging. J. Cosmet. Dermatol..

[78] Cavagnino A., Bobier A., Baraibar M. (2019). The Skin Oxi-Proteome as a Molecular Signature of Exposome Stress. Househ. Pers. Care Today.

[79] Cavagnino A., Starck A., Bobier A., Baraibar M.A. (2022). Protein Carbonylation as a Reliable Read-Out of Urban Pollution Damage/Protection of Hair Fibers. Cosmetics.

[80] Ablon G., Kogan S. (2018). A Six-Month, Randomized, Double-Blind, Placebo-Controlled Study Evaluating the Safety and Efficacy of a Nutraceutical Supplement for Promoting Hair Growth in Women with Self-Perceived Thinning Hair. J. Drugs Dermatol..

[81] Piquero-Casals J., Saceda-Corralo D., Aladren S., Bustos J., Fernández-Botello A., Navasa A., Logusso G., Jourdan E., Mir-Bonafé J.F., Morgado-Carrasco D. (2025). Oral Supplementation with L-Cystine, *Serenoa repens*, *Cucurbita pepo*, and *Pygeum africanum* in Chronic Telogen Effluvium and Androgenetic Alopecia: A Double-Blind, Placebo-Controlled, Randomized Clinical Study. Ski. Appendage Disord..

[82] Ablon G. (2015). A 3-Month, Randomized, Double-Blind, Placebo-Controlled Study Evaluating the Ability of an Extra-Strength Marine Protein Supplement to Promote Hair Growth and Decrease Shedding in Women with Self-Perceived Thinning Hair. Dermatol. Res. Pract..

[83] Thiboutot D., Harris G., Iles V., Cimis G., Gilliland K., Hagari S. (1995). Activity of the Type 1 5 Alpha-Reductase Exhibits Regional Differences in Isolated Sebaceous Glands and Whole Skin. J. Investig. Dermatol..

[84] Jodynis-Liebert J., Kujawska M. (2020). Biphasic Dose-Response Induced by Phytochemicals: Experimental Evidence. J. Clin. Med..

[85] Calabrese E.J. (2020). Stimulating Hair Growth via Hormesis: Experimental Foundations and Clinical Implications. Pharmacol. Res..

[86] Murata K., Noguchi K., Kondo M., Onishi M., Watanabe N., Okamura K., Matsuda H. (2013). Promotion of Hair Growth by *Rosmarinus officinalis* Leaf Extract. Phytother. Res..

[87] Pazoki-Toroudi H., Ajami M., Babakoohi S., Khaki L., Habibey R., Akhiani M., Seirafi H., Firooz A. (2010). Effects of Diphencyprone on Expression of Bcl-2 Protein in Patients with Alopecia Areata. Immunopharmacol. Immunotoxicol..

[88] El-Domyati M., Attia S., Saleh F., Bassyouni M., Barakat M., Abdel-Wahab H. (2010). Evaluation of Apoptosis Regulatory Markers in Androgenetic Alopecia. J. Cosmet. Dermatol..

[89] Marčetić M., Bufan B., Drobac M., Antić Stanković J., Arsenović Ranin N., Milenković M.T., Božić D.D. (2025). Multifaceted Biological Properties of Verbascoside/Acteoside: Antimicrobial, Cytotoxic, Anti-Inflammatory, and Immunomodulatory Effects. Antibiotics.

[90] Andrade J.M., Faustino C., Garcia C., Ladeiras D., Reis C.P., Rijo P. (2018). *Rosmarinus officinalis* L.: An Update Review of Its Phytochemistry and Biological Activity. Future Sci. OA.

[91] Hou C., Miao Y., Wang J., Wang X., Chen C.-Y., Hu Z.-Q. (2015). Collagenase IV Plays an Important Role in Regulating Hair Cycle by Inducing VEGF, IGF-1, and TGF-β Expression. Drug Des. Dev. Ther..

[92] Li H., He H., Liu C., Akanji T., Gutkowski J., Li R., Ma H., Wan Y., Wu P., Li D. (2022). Dietary Polyphenol Oleuropein and Its Metabolite Hydroxytyrosol Are Moderate Skin Permeable Elastase and Collagenase Inhibitors with Synergistic Cellular Antioxidant Effects in Human Skin Fibroblasts. Int. J. Food Sci. Nutr..

[93] Wang J., Yuan M., Li Q., Shen C., Zhang X., Zhu C., Cen Q. (2025). Combined Protection against UVB-Induced Photoaging by Oleuropein, Hydroxytyrosol, and Verbascoside through Modulation of Inflammation, Oxidative Stress, and Collagen Homeostasis. Sci. Rep..

[94] Shin E.J., Lee J.S., Hong S., Lim T.-G., Byun S. (2019). Quercetin Directly Targets JAK2 and PKCδ and Prevents UV-Induced Photoaging in Human Skin. Int. J. Mol. Sci..

