# Supplementary material for: Hair Growth-Supporting and Follicle-Protective Potential of a Botanical-Based Supplement Ingredient: In Vitro, Ex Vivo, and Molecular Docking Studies
Source: Biomolecules. 2026 Aug 18;16(8):1207. doi: 10.3390/biom16081207 (PMC13511542; doi:10.3390/biom16081207)
Supplement: Supplementary file 1 [file biomolecules-16-01207-s001.zip › biomolecules-4465045-supplementary.pdf]

Article

Hair Growth-Supporting and Follicle-Protective Potential of a Botanical-Based Supplement Ingredient: In Vitro, Ex Vivo, and Molecular Docking Studies

Supporting Information

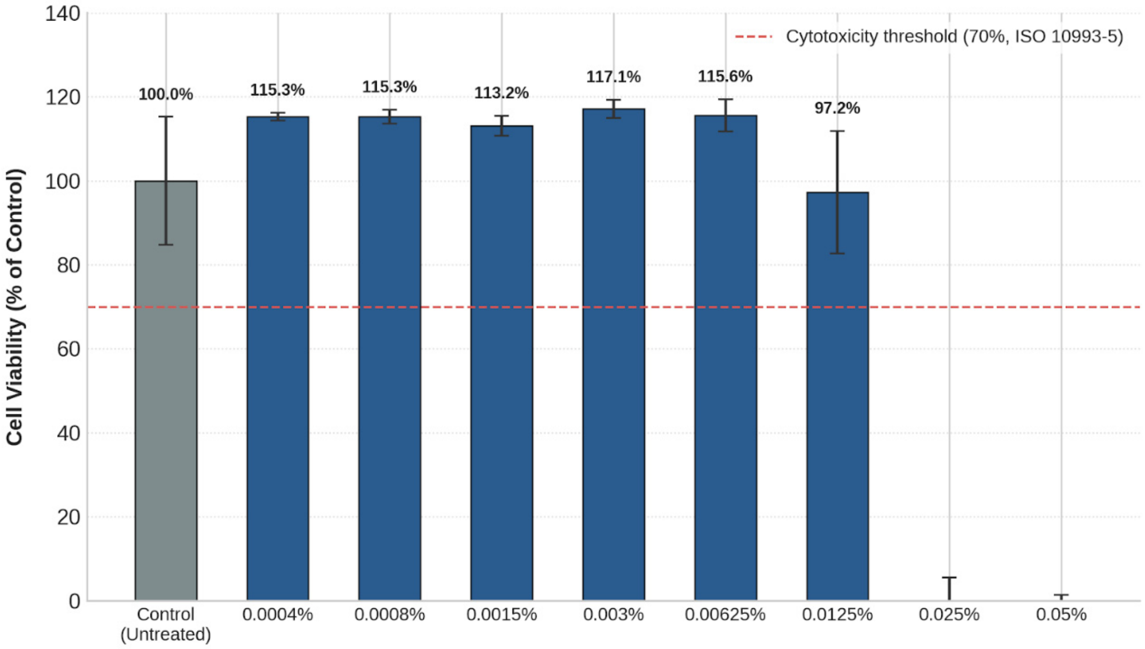

Figure S1. Effect of Elissara on human follicle dermal papilla cell (HFDPC) viability

Cell viability was evaluated using the PrestoBlue/MTT assay following 24 h of exposure to increasing concentrations of Elissara (0.0004%–0.05% w/v). Bar graphs represent cell viability (in blue for tested concentrations and gray for the untreated control) expressed as percentage relative to the control group (100%). Data represents the mean ± standard deviation (SD) of technical quintuplicates (n = 5). The red dashed line denotes the 70% cell viability threshold for biocompatibility according to ISO 10993-5:2009 guidelines. Elissara maintained optimal cell viability (97.25%) at concentrations up to 0.0125%.
